# Selection and characterization of ultrahigh potency designed ankyrin repeat protein inhibitors of *C*. *difficile* toxin B

**DOI:** 10.1371/journal.pbio.3000311

**Published:** 2019-06-24

**Authors:** Rudo Simeon, Mengqiu Jiang, Ana M. Chamoun-Emanuelli, Hua Yu, Yongrong Zhang, Ran Meng, Zeyu Peng, Joanita Jakana, Junjie Zhang, Hanping Feng, Zhilei Chen

**Affiliations:** 1 Department of Microbial Pathogenesis and Immunology, Texas A&M University Health Science Center, College Station, Texas, United States of America; 2 Department of Biochemistry and Biophysics, Texas A&M University, College Station, Texas, United States of America; 3 Department of Microbial Pathogenesis, University of Maryland Dental School, Baltimore, Maryland, United Sates of America; 4 National Center for Macromolecular Imaging, Verna and Marrs McLean Department of Biochemistry and Molecular Biology, Baylor College of Medicine, Houston, Texas, United States of America; Harvard Medical School, UNITED STATES

## Abstract

*Clostridium difficile* infection (CDI) is a major nosocomial disease associated with significant morbidity and mortality. The pathology of CDI stems primarily from the 2 *C*. *difficile*–secreted exotoxins—toxin A (TcdA) and toxin B (TcdB)—that disrupt the tight junctions between epithelial cells leading to the loss of colonic epithelial barrier function. Here, we report the engineering of a series of monomeric and dimeric designed ankyrin repeat proteins (DARPins) for the neutralization of TcdB. The best dimeric DARPin, DLD-4, inhibited TcdB with a half maximal effective concentration (EC_50_) of 4 pM in vitro, representing an approximately 330-fold higher potency than the Food and Drug Administration (FDA)-approved anti-TcdB monoclonal antibody bezlotoxumab in the same assay. DLD-4 also protected mice from a toxin challenge in vivo. Cryo-electron microscopy (cryo-EM) studies revealed that the 2 constituent DARPins of DLD-4–1.4E and U3—bind the central and C-terminal regions of the delivery domain of TcdB. Competitive enzyme-linked immunosorbent assay (ELISA) studies showed that the DARPins 1.4E and U3 interfere with the interaction between TcdB and its receptors chondroitin sulfate proteoglycan 4 (CSPG4) and frizzled class receptor 2 (FZD2), respectively. Our cryo-EM studies revealed a new conformation of TcdB (both apo- and DARPin-bound at pH 7.4) in which the combined repetitive oligopeptides (CROPS) domain points away from the delivery domain. This conformation of the CROPS domain is in stark contrast to that seen in the negative-stain electron microscopy (EM) structure of TcdA and TcdB at the same pH, in which the CROPS domain bends toward and “kisses” the delivery domain. The ultrapotent anti-TcdB molecules from this study serve as candidate starting points for CDI drug development and provide new biological tools for studying the pathogenicity of *C*. *difficile*. The structural insights regarding both the “native” conformation of TcdB and the putative sites of TcdB interaction with the FZD2 receptor, in particular, should help accelerate the development of next-generation anti–*C*. *difficile* toxin therapeutics.

## Abstract 2

This study reports the engineering of designed ankyrin repeat proteins (DARPins) with potent neutralization activity against the secreted exotoxin B from Clostridium difficile, elucidating their mechanism of action by cryo-electron microscopy.

## Introduction

*Clostridium difficile* is a gram-positive spore-forming anaerobic bacterium. Colonization of the gut with pathogenic *C*. *difficile* can lead to *C*. *difficile* infection (CDI) with symptoms including diarrhea, pseudomembranous colitis, sepsis, multiple organ dysfunction syndrome, and even death [1,2]. In 2011, there were almost half a million reported cases of CDI and more than 29,000 CDI-associated deaths in the United States alone [3]. *C*. *difficile* is considered a major nosocomial pathogen because a significant percentage (7%) patients acquire CDI after hospitalization [4]. Broad-spectrum antibiotics are considered a major culprit of CDI, because they disrupt the patients’ natural gut microflora that would otherwise keep the proliferation of *C*. *difficile* in check. The current standard of care for treating CDI is the administration of additional antibiotics, mainly vancomycin, metronidazole, and fidaxomicin. Although this approach is generally effective against primary CDI, in recent decades, the rate of CDI recurrence has significantly increased due to the emergence of antibiotic-resistant and so-called hypervirulent strains (15%–35% CDI recurrence in patients after cessation of antibiotic treatment) [5,6].

The pathology of CDI mainly stems from the 2 *C*. *difficile*–secreted exotoxins, toxin A (TcdA) and toxin B (TcdB), that target small GTPases within the host cells, leading to disruption of the tight junctions and loss of colonic epithelial barrier function. The anti-TcdB monoclonal antibody bezlotoxumab (ZINPLAVA) was approved by the Food and Drug Administration (FDA) in 2016 for treating recurrent CDI [7]. The CDI recurrence rate in patients receiving antibiotics together with IV infusion of bezlotoxumab, although lower than those receiving antibiotics alone (26%–28%), remains high at 15% to 17% [8].

Our long-term goal is to develop highly efficacious antitoxin proteins as oral therapeutics that can directly neutralize the toxins in the gut for treating and/or preventing the recurrent of CDI. These antitoxin proteins are based on a designed ankyrin repeat protein (DARPin), a small antibody-mimic binding scaffold that exhibits very high thermostability, resistance to proteases and denaturants, and a very low immunogenicity [9,10]. DARPins that bind a wide range of molecules with pico- to nanomolar affinity have been identified [11–24]. Furthermore, DARPins can be expressed at high levels in *Escherichia coli* (multigram quantities per liter of culture in fermenters) [10,25], enabling DARPins to be produced at potentially very low cost on a large scale.

Combining phage panning and functional screening, a panel of dimeric DARPins with picomolar in vitro TcdB-neutralization potency were identified. The best dimeric DARPin, DLD-4, exhibited a half maximal effective concentration (EC_50)_ of 4 pM and 20 pM against TcdB from *C*. *difficile* strains VPI10463 (ribotype 087) and M68 (ribotype 120), respectively, which is approximately 330-fold and approximately 33-fold more potent than the FDA-approved anti-TcdB monoclonal antibody bezlotoxumab. DARPin DLD-4 was also efficacious in vivo in 2 mouse models against TcdB challenge, pointing to its potential as a next-generation antitoxin biologic for treating CDI and/or preventing its recurrence.

Cryo-electron microscopy (cryo-EM) was employed to elucidate the binding interfaces of TcdB and DLD-4. Guided by this structural information, competitive enzyme-linked immunosorbent assay (ELISA) revealed that the constituent monomeric DARPins of dimeric DLD-4, 1.4E and U3, interfere with the interaction between TcdB and its receptors chondroitin sulfate proteoglycan 4 (CSPG4) and frizzled class receptor 2 (FZD2), respectively. The ultrahigh toxin-neutralization potency of DLD-4 likely stems from its ability to simultaneously block both these receptors from associating with TcdB. Moreover, our cryo-EM study also revealed a novel conformation of DARPin-bound and apo toxin at neutral pH in which the combined repetitive oligopeptides (CROPS) domain points away from the delivery domain. This is in contrast to that seen in the negative-stain electron microscopy (EM) structures of TcdA and TcdB at the same pH in which the CROPS domain extends toward and kisses the delivery domain [26]. We believe that our cryo-EM structures of TcdB, apo and DARPin-bound, represent the native aqueous conformations that will be invaluable for future antitoxin drug development.

## Results

### Selection of monomeric TcdB-neutralizing DARPins

**Fig 1:**
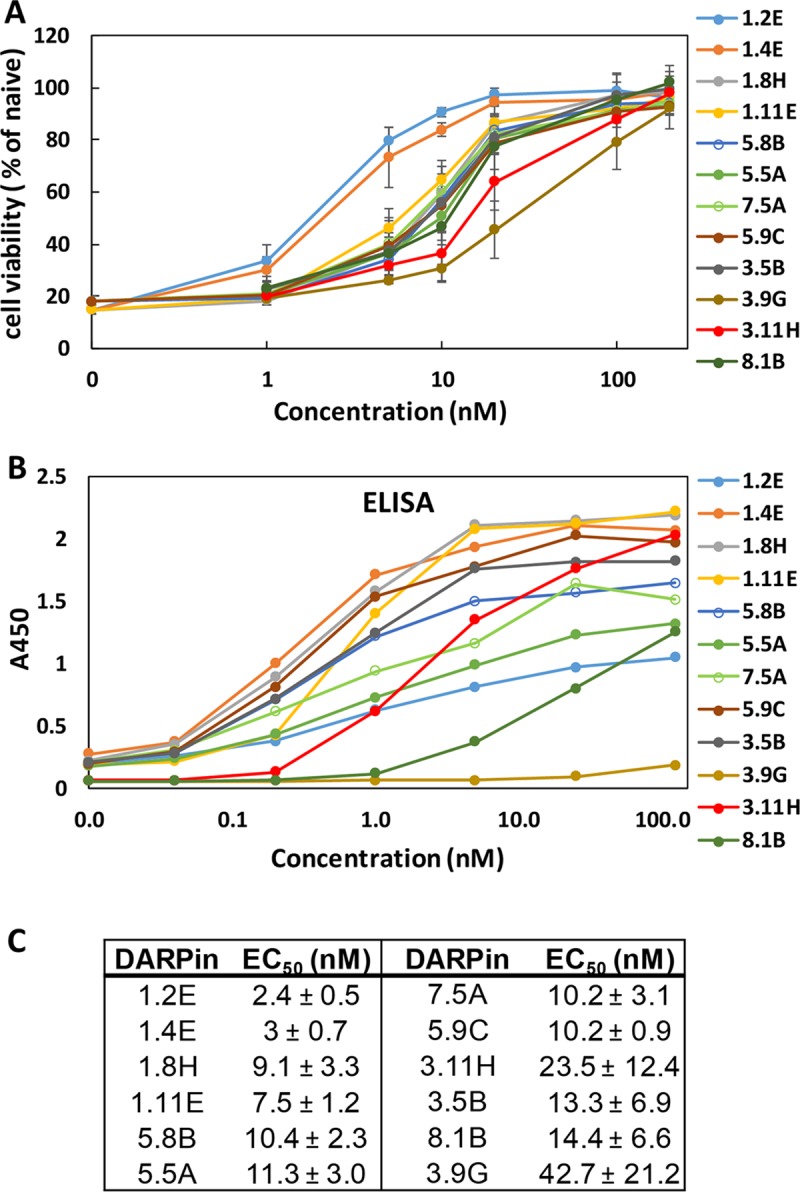
Characterization of leading monomeric DARPin hits. (A) Monomeric DARPins are able to protect Vero cells from TcdB-induced cytopathic effect at nanomolar concentrations. IMAC-purified DARPins were added to Vero cells (2 × 10^3^ cells/well) together with TcdB toxin (5 pg/mL). Cell viability was quantified 72 hours later by the CellTiterGlo assay and normalized to naïve Vero cells. Error bars represent the standard deviation of at least 2 independent experiments performed in duplicate. (B) Relative binding of DARPins to TcdB was determined using ELISA. Serially diluted DARPins were added to microtiter plates coated with 4 μg/mL of TcdB. Results are representative of 2 independent experiments. (C) DARPin monomer TcdB-neutralization potency. Data are the averages of at least 2 independent experiments. DARPin, designed ankyrin repeat protein; EC_50_, half maximal effective concentration; ELISA, enzyme-linked immunosorbent assay; IMAC, Immobilized metal affinity chromatography; TcdB, *C*. *difficile* toxin B.

A library of approximately 2 × 10^9^ DARPin variants was constructed via sequential PCR and ligation essentially as described previously [9]. Biotinylated TcdB (from *C*. *difficile* strain VPI10463) was used as the target protein to enrich DARPins that could bind the toxin via 4 rounds of phage panning. The enriched DARPin library pool from the third round of phage panning were subcloned into the pET26b vector and transformed into *E*. *coli* BL21(DE3) cells for high-level DARPin expression and functional screening for those with toxin-neutralization ability. About 40% of the clones (299 clones) were able to rescue Vero cells’ viability from TcdB toxicity by >50%. The top 40 hits were sequenced, and of these, 12 were determined to be unique clones (Fig 1, S1 Table). Most clones exhibited EC_50_ values of approximately 10 nM, and the 2 best clones, 1.2E and 1.4E, displayed EC_50_ values of 2.4 nM and 3 nM, respectively. The relative affinity of each of the top 9 DARPins for TcdB was assessed by ELISA (Fig 1B) and was found to not directly correlate with their in vitro neutralization potency. For example, although 1.2E and 1.4E neutralized TcdB with similar potency, 1.4E appeared to be among the strongest toxin binders, whereas 1.2E was one of the weaker binders. The discrepancy between binding affinity and neutralization potency likely stems from the different epitopes engaged by the different DARPins. That is, a DARPin that weakly binds a region critical for toxin activity might exhibit a higher toxin-neutralization potency than another DARPin that binds strongly to a region of less importance.

### Selection of dimeric TcdB-neutralizing DARPins

**Fig 2:**
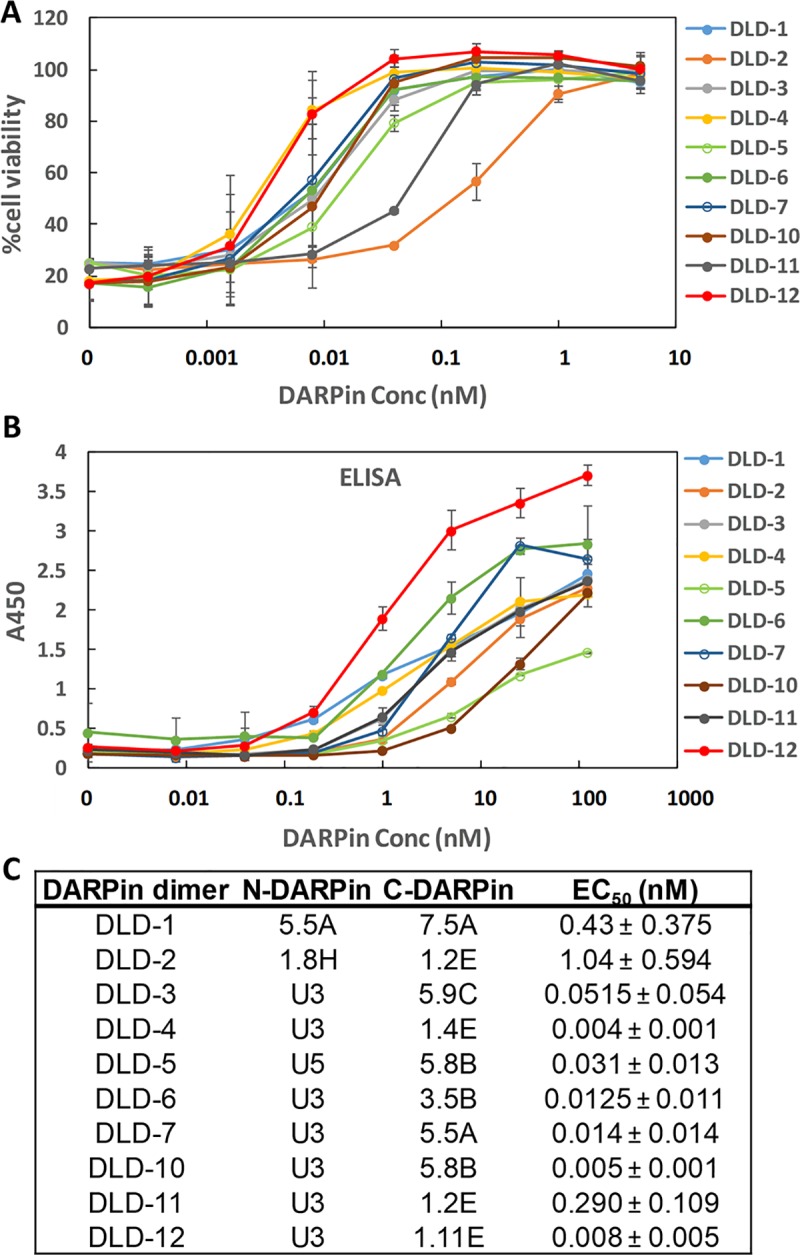
Characterization of dimeric DARPins. (A) Dimeric DARPins protect Vero cells from the TcdB-induced cytopathic effect at picomolar concentrations. IMAC-purified DARPin dimers were added to Vero cells (1.5 × 10^3^ cells/well) together with TcdB toxin (5 pg/mL). Cell viability was quantified 72 hours later by the CellTiterGlo assay and normalized to naïve Vero cells. Error bars represent the standard deviation of 2 independent experiments performed in duplicate. (B) Relative binding of DARPin dimers to TcdB was determined using ELISA. Serially diluted DARPins were added to microtiter plates coated with 4 μg/mL of TcdB. Results are representative of 2 independent experiments, and the error bars represent mean deviation from duplicate samples. (C) TcdB-neutralization potency of DARPin dimers. Data are the averages of at least 2 independent experiments. Conc, concentration; DARPin, designed ankyrin repeat protein; EC_50_, half maximal effective concentration; ELISA, enzyme-linked immunosorbent assay; IMAC, Immobilized metal affinity chromatography; TcdB, *C*. *difficile* toxin B.

**Fig 3:**
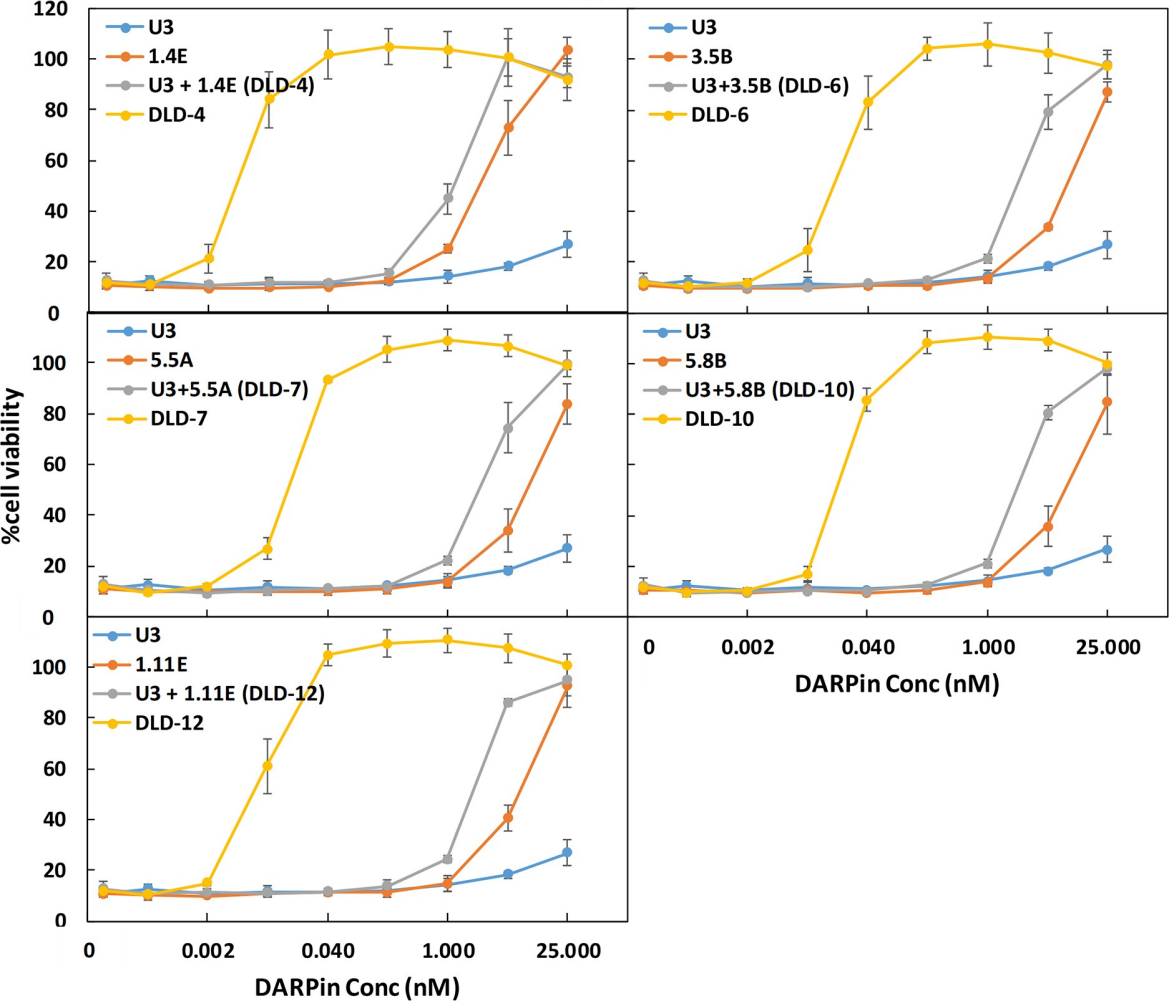
DARPin dimers exhibit superior toxin-neutralization potency relative to the constituent monomers. IMAC-purified DARPin dimers or monomers were added to Vero cells (1.5 × 10^3^ cells/well) together with TcdB (5 pg/mL). Cell viability was quantified 72 hours later by CellTiterGlo assay and normalized to naïve Vero cells. Error bars represent the standard deviation of at least 2 independent experiments performed in duplicate. Conc, concentration; DARPin, designed ankyrin repeat protein; IMAC, Immobilized metal affinity chromatography; TcdB, *C*. *difficile* toxin B.

**Fig 4:**
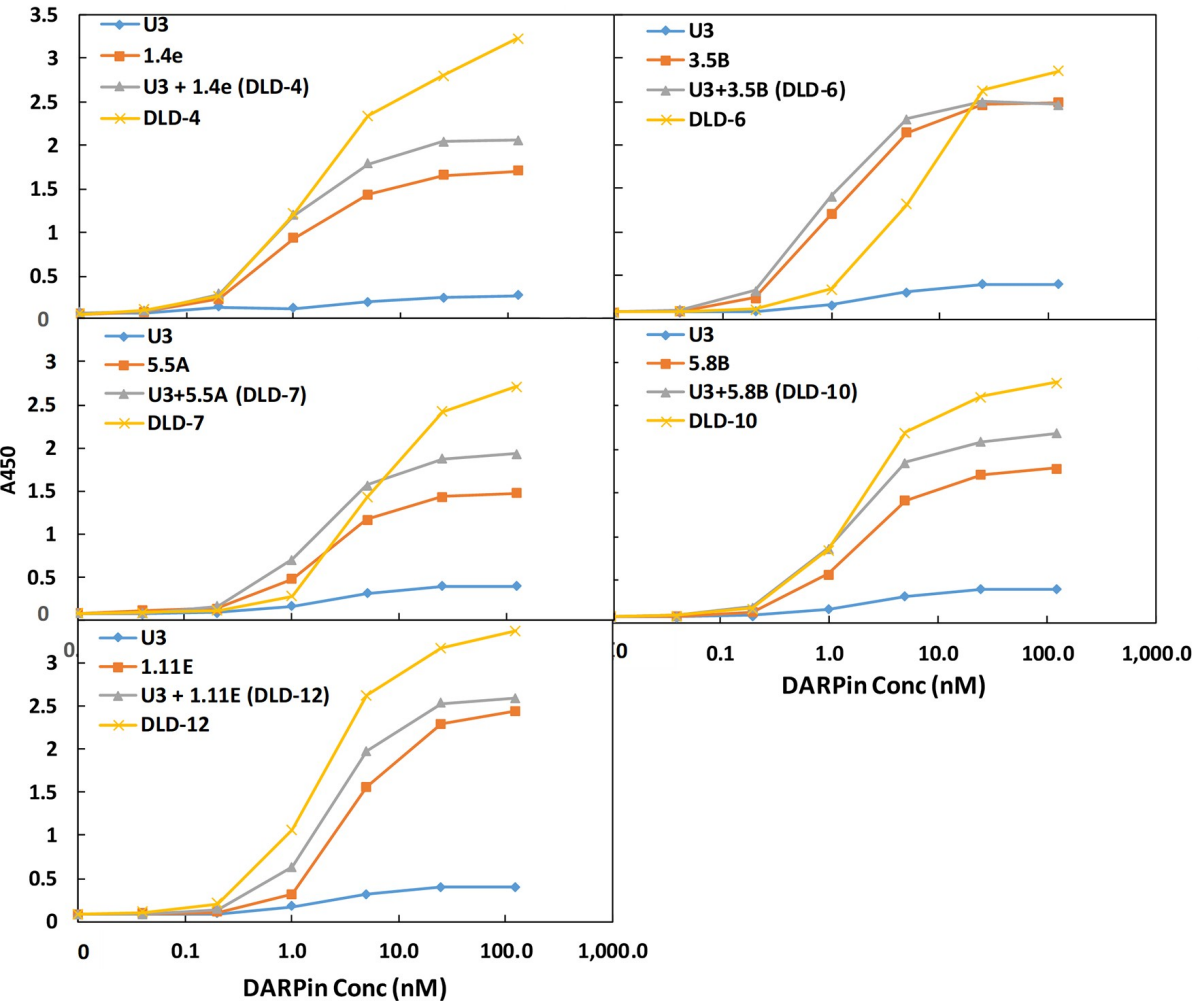
DARPin dimers demonstrate avidity in TcdB binding. Binding of selected DARPin monomers, pooled monomers, or dimers to TcdB was determined using ELISA. Combinations of serially diluted DARPins were added to microtiter plates coated with 4 μg/mL of TcdB. Results are representative of 2 independent experiments. Conc, concentration; DARPin, designed ankyrin repeat protein; ELISA, enzyme-linked immunosorbent assay; TcdB, *C*. *difficile* toxin B.

Fusion of multiple DARPins have been reported to significantly enhance the target-binding affinity via avidity effects [27,28]. We hypothesized that fusion of 2 DARPins that bind nonoverlapping epitopes on the toxin should yield enhanced binding affinity and thus a higher toxin-neutralization potency. A combinatorial library of DARPin dimers was created by joining individual monomeric DARPins (12 total) via a flexible linker (GGGGSx3) (S1 Table). A total of 1,504 individual clones were screened using a Vero cell toxin challenge assay, and 12 hits were identified. Of these, 10 were determined to be unique clones. The in vitro neutralization potencies of these 10 DARPins and their relative TcdB binding affinities are shown in Fig 2. The best DARPin dimer, DLD-4, exhibited a toxin-neutralization EC_50_ of 4 ± 1 pM, which is approximately 600-fold lower than the best monomeric DARPin, 1.2E (EC_50_ 2.4 ± 0.5 nM). As seen with the DARPin monomers, the relative TcdB binding affinities of the DARPin dimers were found to not directly correlate with their neutralization potency. Sequencing results also revealed that many of the dimers contained a new DARPin, U3 or U5, that was not present among the original 12 monomers (Fig 2C, S1 Table). U5 is identical to U3 except that it lacks the third randomized ankyrin repeat (AR) domain. U3 alone exhibits weak, but detectable, toxin-neutralization activity (EC_50_ > 25 nM; Fig 3) in Vero cells and toxin-binding ability (Fig 4). U3 and U5 likely represent a minor constituent of the initial pool used to create the dimer DARPin library, and their discovery is highly serendipitous because (1) they bind to a neutralizing epitope and (2) the U3/U5 neutralization epitope is adjacent to the epitope targeted by the other DARPin hits such that the linker used is of sufficient length to accommodate simultaneous binding to both epitopes.

To understand the reason for the dramatic improvement in activity, we further characterized the 5 dimer DARPins with the strongest toxin-neutralization potency. We first compared the in vitro TcdB-neutralization potency of the DARPin dimers with their constituent monomers (Fig 3). All these DARPin dimers significantly outperformed their individual constituent monomers as well as the combination of both monomers, indicative of synergistic activity. We further compared the relative binding affinity of DARPin dimers and monomers using ELISA. As shown in Fig 4, except for DLD-6, all the dimeric DARPins yielded an increase in the ELISA signal relative to the constitute monomers. This observation is consistent with the binding of nonoverlapping epitopes on the toxin by the monomeric DARPins via an avidity effect. An exception is DLD-6, which appeared to bind the toxin more weakly than its constituent DARPin 3.5B at concentrations <25 nM. This result is somewhat unexpected, considering that the toxin-neutralization potency of DLD-6 is >100-fold higher than that of 3.5B (EC_50_: 12.5 pM for DLD-6 versus 13.3 nM for 3.5B). A sandwich ELISA assay confirmed that DLD-6 lacks the ability to crosslink 2 different TcdB molecules (S1 Fig), which would have been responsible for the difference between neutralization potency and binding result. We posit that the most possible cause of this discrepancy is that TcdB protein may exhibit less flexibility when immobilized on an ELISA plate, thus hindering the simultaneous interaction with both U3 and 3.5B.

### Characterization of TcdB-neutralization potency of DARPins

**Fig 5:**
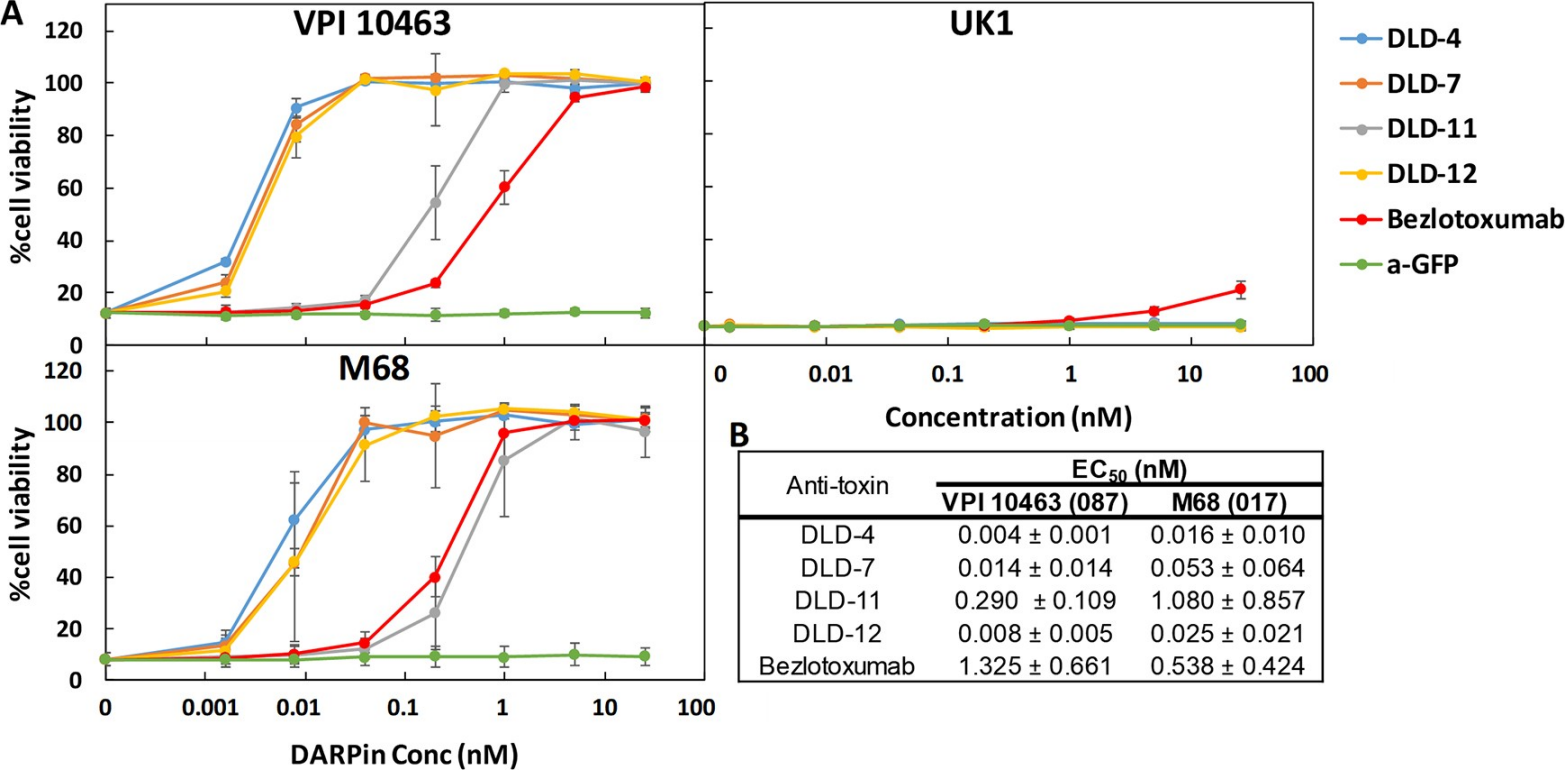
DARPin dimers offer superior protection to Vero cells against the toxicity of TcdB from *C*. *difficile* strains VPI 10463 (ribotype 087) and M68 (ribotype 017). (A) IMAC-purified DARPins were added to Vero cells (1.5 × 10^3^ cells/well) together with TcdB toxin (2.5 pg/mL). Cell viability was quantified 72 hours later by the CellTiterGlo assay and normalized to naïve Vero cells. Error bars represent the standard deviation of at least 2 independent experiments done in duplicate. Bezlotoxumab is the FDA-approved monoclonal antibody for treating recurrent CDI [7]. a-GFP is a GFP-binding DARPin [15] and was used here as a negative control. (B) DARPin dimer TcdB-neutralization potency. Data are the averages of at least 2 independent experiments. CDI, *Clostridium difficile* infection; Conc, concentration; DARPin, designed ankyrin repeat protein; EC_50_, half maximal effective concentration; FDA, Food and Drug Administration; IMAC, Immobilized metal affinity chromatography; TcdB, *C*. *difficile* toxin B.

All of our protein engineering work was conducted using TcdB from the laboratory strain of *C*. *difficile* VPI10463 (ribotype 087). Because there is a significant amount of amino acid sequence heterogeneity between different strains of *C*. *difficile* [29], there is a need to develop broadly neutralizing DARPins. As a first step to address this need, we evaluated the activity of selected DARPin dimers (DLD-4, -7, -11, -12) against TcdB from 3 different strains of *C*. *difficile*: VPI10463, M68 (ribotype 012), and UK1 (ribotype 027). All DARPins were effective against toxins from VPI10463 and M68 (Fig 5). The best DARPin, DLD-4, was found to be approximately 330- and approximately 33-fold more efficacious than bezlotoxumab at inhibiting TcdB from VPI 10463 and M68, respectively, as quantified using the CellTiter-Glo (CTG) assay (Fig 5B). The EC_50_s of DLD-4 and bezlotoxumab were further measured using the conventional cell rounding assay and were found to be similar to that determined using CTG assay (S2 Fig). However, these DARPins showed negligible activity against TcdB from the UK1 strain, which belongs to the hypervirulent 027 ribotype (Fig 5C). Bezlotoxumab also showed significantly weaker, albeit detectable, neutralization activity against this toxin (EC_50_ > 25 nM). We further tested all the other DARPins and found that 5 of them (3.9G, 1.2E, 8.1B, 1.8H, and 1.4E) showed weak but detectable neutralization against TcdB_UK1_ (S3 and S4 Figs). We are currently repeating our in vitro engineering using TcdB_UK1_ as the target protein in order to identify DARPins efficacious against this toxin. To ensure that the specific activity of our bezlotoxumab is not compromised during production, we repeated the EC_50_ measurement of our bezlotoxumab exactly following a published protocol by Orth and colleagues [30] because it is not uncommon for different assays to give different EC_50_ values for the same reagent. The EC_50_ of bezlotoxumab was found to be 0.3 nM, which is nearly identical to that reported by Orth and colleagues using the authentic bezlotoxumab produced by Merck, confirming the integrity of our in-house–produced bezlotoxumab.

**Fig 6:**
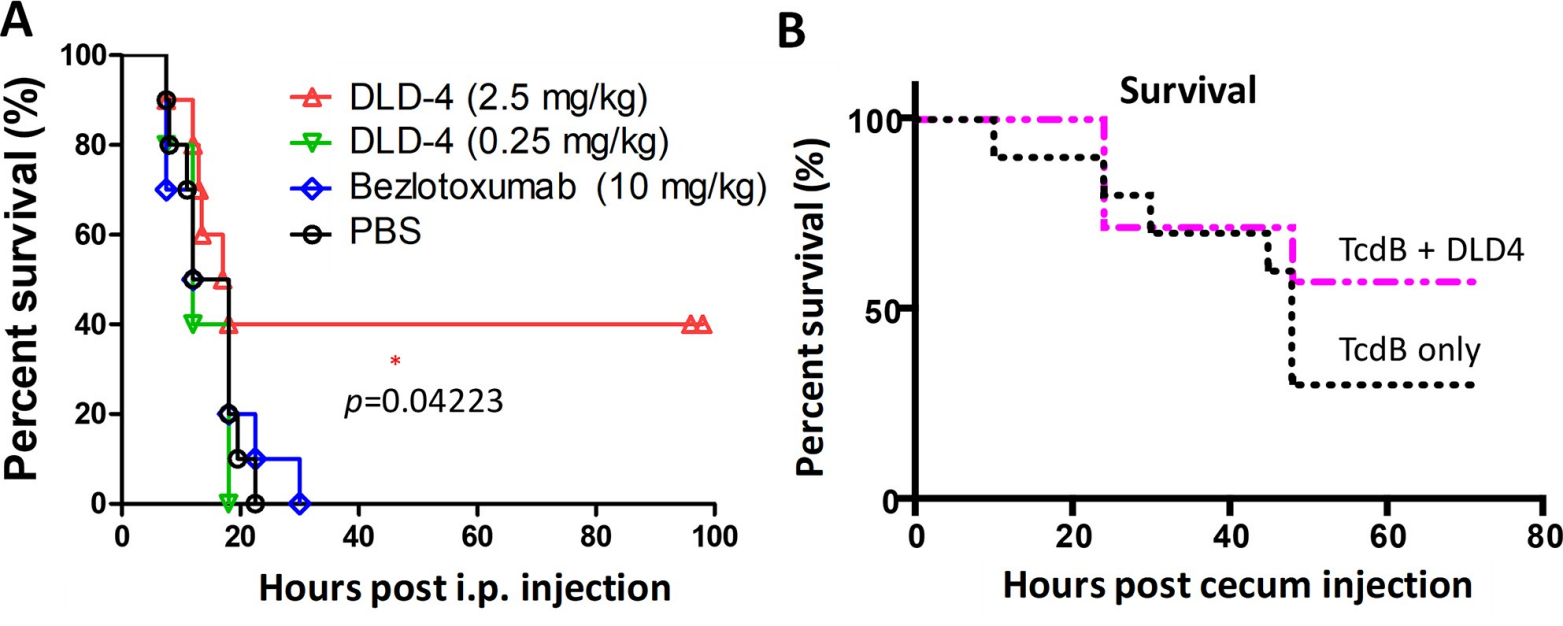
In vivo studies. (A) Mice were IP injected with TcdB (1.5 μg/kg) alone (PBS, *n* = 10) or together with DLD-4 (0.25 or 2.5 mg/kg, *n* = 10) or bezlotoxumab (10 mg/kg, *n* = 10). Mouse survival rate was monitored, and data were analyzed by Kaplan–Meier survival analysis with log-rank test of significance; *p* = 0.04 (DLD-4 versus PBS). (B) DLD-4 (5 mg/mouse) was mixed with TcdB (15 μg/mouse) in 100 μL PBS and injected immediately into the cecum of mice (*n* = 7). The control group received the same dose of TcdB alone (*n* = 10). Mouse survival was monitored for 3 days, and the data were analyzed by Kaplan–Meier survival analysis; *p* = 0.349 (TcdB alone versus TcdB + DLD-4). IP, intraperitoneally; TcdB, *C*. *difficile* toxin B.

We further evaluated the ability of the most potent antitoxin DARPin, DLD-4, to protect mice from systemic toxin challenge in vivo using 2 murine TcdB challenge models. In the first model, a lethal dose of TcdB (1.5 μg/kg) was mixed with DLD-4 (0.25 or 2.5 mg/kg), bezlotoxumab (10 mg/kg), or PBS and then injected intraperitoneally (IP) into CD1 mice (5–10 mice/group) [31,32]; 40% of the mice survived after injection with the mixture containing the toxin mixed with 2.5 mg/kg DLD-4 (*p* = 0.04; Fig 6A). These results suggest that DLD-4 possesses significant toxin-neutralization ability in vivo. Bezlotoxumab at 10 mg/kg dose did not show any protection against TcdB in this experiment nor in experiments in which lower dosages of TcdB were used (S5 Fig). This result is not particularly surprising considering that, in the phase III clinical trial, bezlotoxumab did not increase the initial clinical cure rate of CDI [8] and was only approved by the FDA for reducing the recurrence of CDI, not as a treatment. We did not attempt the spore challenge model [33] because we wished to decouple in vivo antitoxin activity from half-life, and—unlike antibodies—unmodified DARPins suffer from a very short circulation half-life in vivo due to their small size [34].

The second model is the murine cecum injection model as described previously [35]. A minor survival advantage (not statistically significant) was observed for mice receiving TcdB and DLD-4 compared with TcdB alone (Fig 6B). Further studies revealed that the less-than-expected in vivo efficacy of DLD-4 in this cecum injection model was most likely due to its poor protease stability. Specifically, DARPin DLD-4 is sensitive to digestion by trypsin and chymotrypsin (S6 Fig), the most abundant protease in the gastrointestinal tract [36,37]. We are currently engineering second-generation trypsin- and chymotrypsin-stable variants of DLD-4.

### Cryo-EM structure of the full-length TcdB and its interaction with DARPin DLD-4

To understand how DLD-4 neutralizes TcdB, single-particle cryo-EM was used to elucidate the complex structure of TcdB and DLD-4. Purified full-length TcdB (VPI 10463) and DLD-4 were mixed and vitrified on the cryo-EM grids and then imaged under cryo-EM. A total of 3,902 super-resolution electron-counting movie stacks were obtained, from which 84,420 good image particles were used to reconstruct the final 3D density map with an overall resolution of 9 Å (see Materials and methods, S7 and S8 Figs).

The overall structure of full-length TcdB is very similar to TcdA [38], consisting of 4 functional domains: the glucosyltransferase domain (GTD), the autoprocessing domain (APD), the delivery domain, and the CROPS domain. The delivery domain undergoes conformational changes triggered by the low-pH environment in the late endosome to form pores [39,40], translocating the GTD across the endosomal membrane into the host cytosol following the APD-catalyzed intramolecular cleavage reaction [41,42]. Once inside the cytosol, the GTD inactivates small GTPases such as Ras homolog gene family, member A (RhoA), Rac family small GTPase 1 (Rac1), and cell division cycle 42 (Cdc42) by glucosylation [43,44], causing a loss of actin polymerization and cytoskeletal changes and disruption of the colonic epithelial junctions.

**Fig 7:**
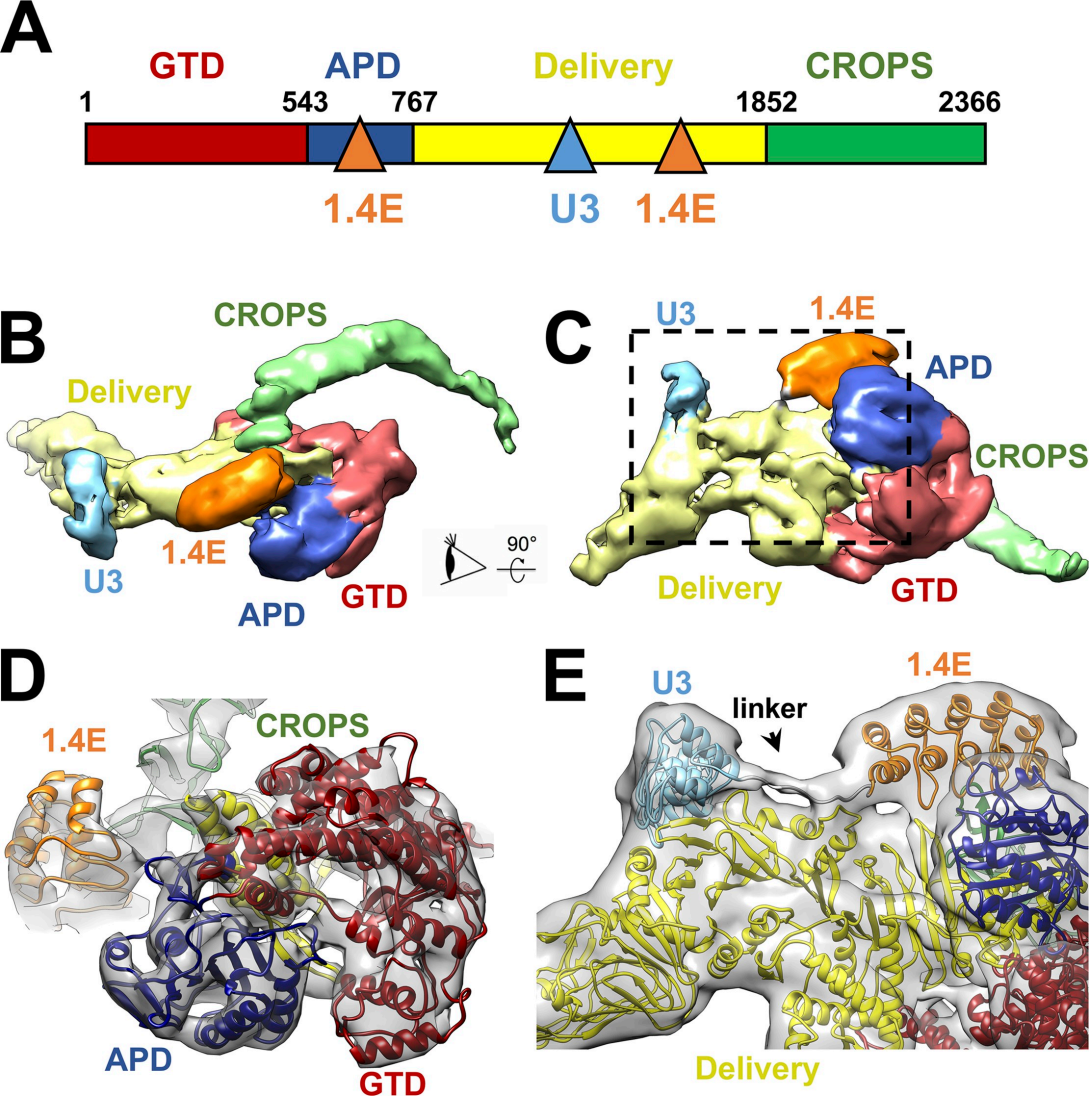
Cryo-EM structure of the TcdB–DLD-4 complex. (A) Domain organization of TcdB and its interaction with the 2 modules of the DLD-4 DARPin. The binding sites on TcdB for DLD-4 are indicated by the blue and orange triangles for the U3 and 1.4E modules, respectively. (B) Density map of the TcdB–DLD-4 complex with the functional domains and DARPin modules in different colors. (C) The same map but rotated 90° as indicated by the arrow. (D) The model fitted into the map as viewed from the eye cartoon labeled in Panel B. The density map is iso-surfaced at a threshold of 9 σ. (E) Zoom-in of the region labeled within the dashed black box in Panel C to show the linker between DARPins U3 and 1.4E. The density map is iso-surfaced at a threshold of 3.8 σ. APD, autoprocessing domain; CROPS, combined repetitive oligopeptides; cryo-EM, cryo-electron microscopy; DARPin, designed ankyrin repeat protein; GTD, glucosyltransferase domain; TcdB, *C*. *difficile* toxin B.

A 3D classification of all the cryo-EM particles of TcdB and DLD-4 revealed multiple conformations of the CROPS domain (S7 Fig), indicating a continuum of conformational variations at the C-terminal tip of the CROPS domain. In all observed conformations, the CROPS domain projects away from the delivery domain (Fig 7B and S8 Fig). This conformation of the CROPS domain is strikingly different from that reported in the negative-stain EM structure of the full-length apo TcdA and TcdB at the same pH (pH 7.4), in which the CROPS domain extends toward and “kisses” the delivery domain [45]. This observation prompted us to determine the cryo-EM structure of apo TcdB. The CROPS domain of apo TcdB at pH 7.4 was found to adopt exactly the same conformation as those in complex with DARPin (S7 Fig). To resolve whether the 180° shift of the CROPS domain results from the difference between cryo-EM and negative-staining EM specimen preparations, we calculated the 2D class averages of the negatively stained apo TcdB particles at pH 7.4. In approximately 80% of the negatively stained TcdB particles, the CROPS domain extends toward the delivery domain (S9 Fig), as seen in the negatively stained apo TcdA and TcdB. Only approximately 20% of the apo TcdB particles have the CROPS domain projecting away from the delivery domain similar to that observed in the cryo-EM TcdB structure. Unlike cryo-EM, in which the protein samples are preserved in a vitreous state with water molecules in and surrounding the specimen [46], negative-stain EM inevitably results in dehydration and flattening of the biological specimens, which may result in distortion of the molecule’s conformation. Thus, we believe that the TcdB conformation in which the CROPS domain projects away from, rather than extends toward, the delivery domain represents the native aqueous conformation of TcdB at pH 7.4 (S10 Fig).

The 2 constituent DARPins of DLD-4, U3 and 1.4E, bind around the middle and the C-terminal end of the delivery domain, respectively (Fig 7A, 7B and 7C). DARPin 1.4E also interacts with the C-terminal region of the APD. The resolved protein secondary structures allowed us to model each domain of the TcdB and DLD-4 into the cryo-EM density (Fig 7D). The density of the 15-residue–long peptide linker between the C-terminus of U3 and the N-terminus of 1.4E was visible, enabling the assignment of each DARPin into its corresponding density (Fig 7E). The local resolutions of the cryo-EM density are not uniform throughout the complex, with lower resolutions around U3 of DLD-4 and the C-terminal tip of the CROPS domain. This is consistent with the observation that DARPin U3 binds relatively weakly to TcdB, and the tip of the CROPS domain exhibits relatively large flexibility, properties that contribute to lower local resolutions in single-particle cryo-EM reconstructions [47]. We further noticed that the protruding tip of the delivery domain in both DLD-4-bound and apo TcdB seems to bend more toward the center of the toxin than that seen in the crystal structure of apo TcdA [38] (S1 Movie and S11 Fig), which may reflect an intrinsic difference between TcdB and TcdA or compaction of TcdA in the crystal lattice.

**Fig 8:**
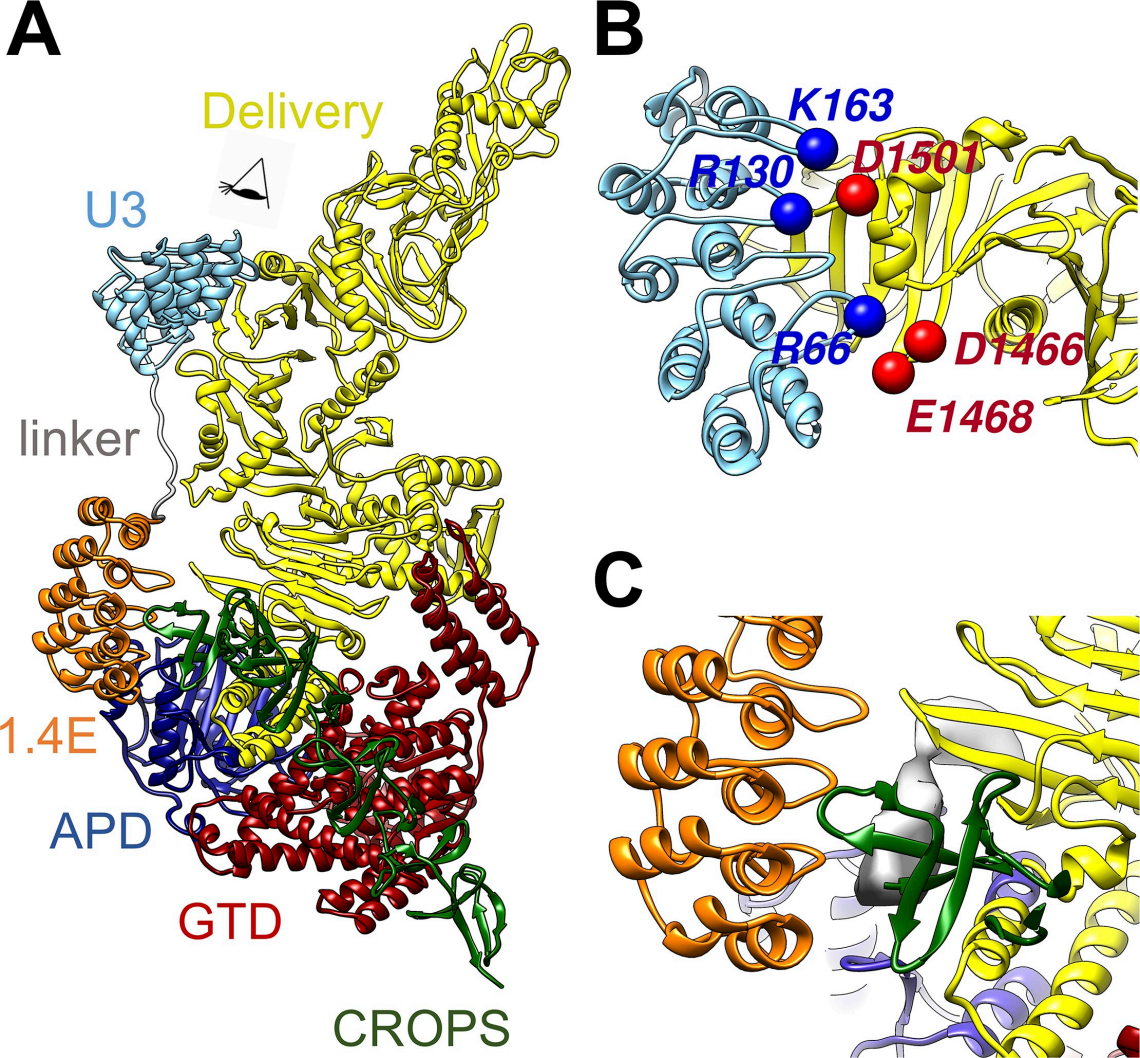
Interactions between TcdB and DLD-4. (A) Overall model of the complex. (B) Zoom-in view to show the interactions between U3 DARPin and TcdB as viewed in the direction labeled by an eye cartoon in Panel A. (C) Zoom-in view to show the interactions between the DARPin 1.4E and TcdB at the region as labeled by red dashed box in Panel A. APD, autoprocessing domain; CROPS, combined repetitive oligopeptides; DARPin, designed ankyrin repeat protein; GTD, glucosyltransferase domain; TcdB, *C*. *difficile* toxin B.

Examination of the interface between TcdB and DLD-4 revealed detailed binding modes between the toxin and the 2 constituent DARPins, U3 and 1.4E, which are separated by approximately 30 Å and orientated perpendicular to each other (Fig 8A). The 2 DARPins are connected by a 15-residue linker (GGGGSx3), which lies along the side of the delivery domain of TcdB (Fig 7E). Each DARPin consists of 3 designed AR modules sandwiched between the N- and C-terminal capping ARs [9,10]. Each designed AR and the C-terminal capping AR consist of a β-turn loop followed by 2 antiparallel α-helices, resulting in 3 variable loops and 1 fixed loop on each DARPin. These 4 loops are labeled as loops 1–4 in Fig 8. As anticipated, both DARPins have their variable loop regions contacting the TcdB and their helical scaffold regions exposed to the solvent. U3 grips onto the middle of the delivery domain (a β-sheet from residues 1,461–1,510 and a loop from residues 1,595–1,603, Fig 8B), whereas 1.4E sits between the C-terminus of the delivery domain and the N-terminus of the CROPS domain (residues 1,800–1,834) and also interacts with a loop from the APD (residues 747–751; Fig 8C). The sequence of TcdB from 1,800 to 1,834 lacks a high-resolution structural reference. However, both the shape of the EM density and the secondary structure prediction based on the protein sequence suggest that this region is a β-sheet (gray density in Fig 8C and S12 Fig).

**Fig 9:**
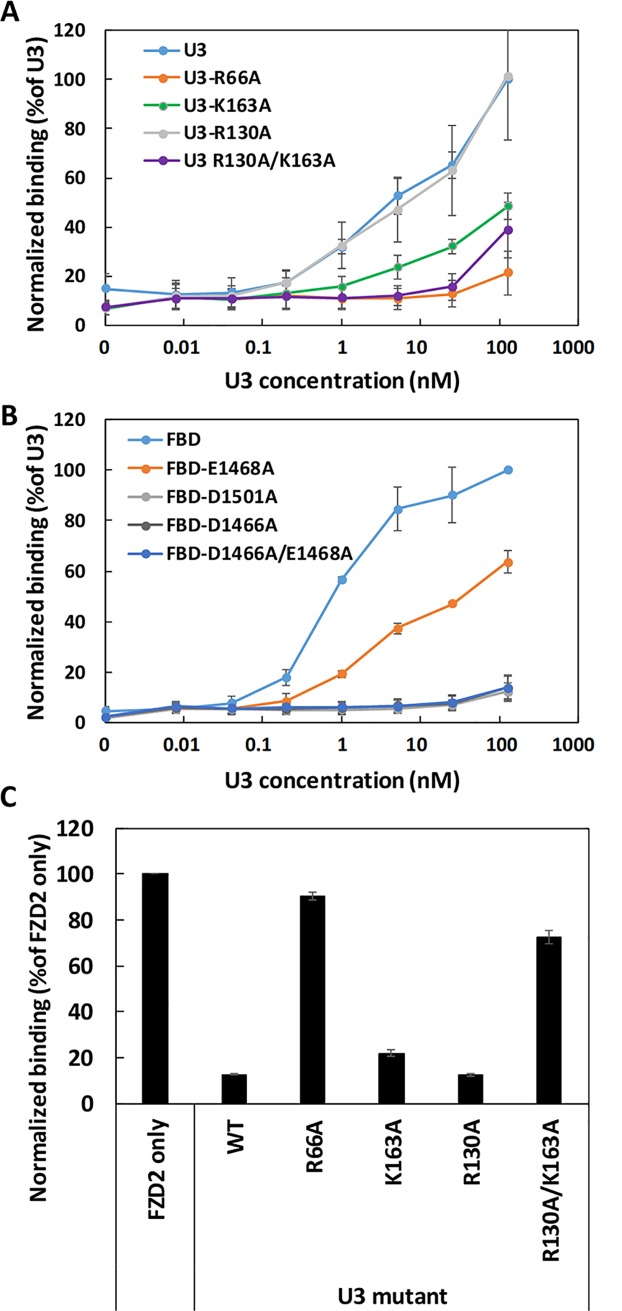
Charge contribution of binding between U3 and TcdB_VPI_. Relative binding of U3 to different immobilized mutants of FBD (A) or different U3 mutants to immobilized TcdB_VPI_ (B) was determined using ELISA. In both cases, serially diluted WT or mutant U3 proteins were added to microtiter plates coated with 4 μg/mL of the TcdB_VPI_ (A) or FBD variants (B). Results are the average of 3 independent experiments, and the error bars represent the standard deviation. (C) Relative binding of FZD2-Fc to immobilized TcdB_VPI_. The ELISA plates were coated with TcdB_VPI_ followed by treatment with 1 nM of FZD2-Fc alone or in combination with 250 nM of WT or different U3 mutants. The error bars represent mean deviation from 2 independent experiments. ELISA, enzyme-linked immunosorbent assay; FBD, frizzled binding domain; FZD2-Fc, Fc-tagged extracellular domain of FZD2; TcdB, *C*. *difficile* toxin B; WT, wild type.

The cryo-EM structure revealed several protein residues with opposing charges at the binding interface between DLD-4 and TcdB. For example, arginine 66 on Loop 1 in U3 is within a Cα-Cα distance of 5 Å to glutamic acid 1,468 and aspartic 1,466, while arginine 130 (Loop 3) and lysine 163 (Loop 4) in U3 are facing aspartic acid 1,501 in the TcdB delivery domain (Fig 8B). To study the effect of interacting residues, U3 and frizzled binding domain (FBD; amino acids 1,285–1,804) of TcdB_VPI_ [48] mutants each harboring specific mutations were constructed and purified. Mutation arginine 66 alanine greatly impaired the ability of U3 to bind or neutralize TcdB_VPI_. (Fig 9A and S13 Fig). U3_R66A_ also did not appreciably block FZD2 binding to immobilized TcdB_VPI_ (Fig 9C). Conversely, FBD with the mutation aspartic acid 1466 alanine (FBD_D1466A_) exhibited negligible binding to U3, whereas FBD_E1468A_ showed significantly reduced binding affinity to U3 (Fig 9B). These results indicate that both the negatively charged residues glutamic acid 1468 and aspartic acid 1466 on TcdB_VPI_ may interact with the positively charged arginine 66 on U3, with aspartic acid 1466 playing a more dominant role (Fig 9A). Similarly, FBD_D1501A_ showed negligible ability to bind U3, whereas U3_K163A_ showed significantly weaker affinity for TcdB_VPI_. Mutant U3_R130A_ retained similar affinity for TcdB_VPI_ relative to U3, but the double-mutant U3_R130A/K163A_ showed further reduced binding affinity to TcdB_VPI_ relative to the single-mutant U3_K163A_. The double-mutant U3_R130A/K163A_ also exhibited a weaker ability to block the binding of FZD2 to immobilized TcdB_VPI_ than either of the single mutants U3_R130A_ and U3_R163A_ via ELISA (Fig 9C). These results indicate the presence of important ionic interaction between the negatively charged residue aspartic acid 1501 on TcdB_VPI_ and the positively charged residues lysine 163 and arginine 130 on U3, with lysine 163 being the dominant contributor on U3. In TcdB_UK1_, the negatively charged glutamic acid 1468 and aspartic acid 1501 are occupied by the positively charged lysine and polar asparagine, respectively (residues labeled in orange in S12A Fig). The differences in change at these residues likely explains the weakened affinity of U3 toward TcdB_UK1_ (S14 Fig).

For DARPin 1.4E, the loops 1 and 2 span over a β-sheet region (residues 1,749–1,767) in the TcdB delivery domain, whereas loops 3 and 4 interact with part of the CROPS domain (residues 1,800–1834) and the APD (residues 747–751), respectively. Mutation of the charged residues glutamic acid 144 and lysine 168 in 1.4E to alanine had minor effect on the ability of 1.4E to interact with TcdB_VPI_ (S13 Fig), indicating that charge interaction mediated by these residues likely does not play a significant role in 1.4E binding to TcdB. Unfortunately, the region on TcdB that interfaces with 1.4E, spanning residues 1,800 to 1,834, lacks a high-resolution structural reference, making it difficult to identify specific interactions with reasonable confidence. It is worth noting that the region of residues 1,753 to 1,851 was reported to be highly variable between TcdB from different ribotypes, and neutralizing epitopes within this region were found to be less accessible in TcdB of ribotype 027 (e.g., UK1) than that of ribotype 012 [49]. No detectable binding between 1.4E and the UK1 strain of TcdB was observed in our ELISA assay (S14 Fig). Thus, the lack of neutralization activity of 1.4E against TcdB from UK1 may be in part due to the occlusion of the neutralization epitope in this toxin.

### Mechanism of TcdB neutralization by DARPin DLD-4

Both TcdA and TcdB enter cells via receptor-mediated endocytosis [50–52]. The CROPS domain is historically thought to be the sole receptor-binding domain [30,53]. However, 3 cell-surface receptors for TcdB have recently been reported: CSPG4, poliovirus receptor like 3 (PVRL3 or NECTIN3), and members of the frizzled protein family (FZD1, FZD2, and FZD7) [54–56]. The constituent monomer DARPins in the dimer DLD-4 are 1.4E and U3. The cryo-EM study revealed that 1.4E interacts with regions in TcdB between residues 747 and 751 and 1,800 and 1,834, whereas U3 interacts with regions between residues 1,461 and 1,510 and 1,595 and 1,603 (Fig 7A). In vitro studies showed that, although 1.4E binds to the APD of TcdB, which has been implicated in inositol hexaphosphate (IP6)-induced autocleavage, the DARPin exhibits negligible impact on the IP6-induced toxin autocleavage activity (S15 Fig). The overlap of the 1.4E-interacting regions with that of the binding site of TcdB receptor CSPG4 (between residues 1,756 and 1,852, designated D97) [57] led us to hypothesize that 1.4E may exert its toxin-neutralization activity by interfering with the toxin–CSPG4 interaction. Similarly, alignment of our cryo-EM structure of TcdB–DARPin complex with the crystal structure of the TcdB and the frizzled receptor-binding domain revealed a complete overlap of U3 and FZD2 on TcdB [48] (S16 Fig), indicating that U3 likely neutralizes TcdB by interfering with its interaction with FZD1/2/7. We set about testing these hypotheses as well as investigating the potential disruption of the toxin’s interaction with other known receptors by DLD-4.

**Fig 10:**
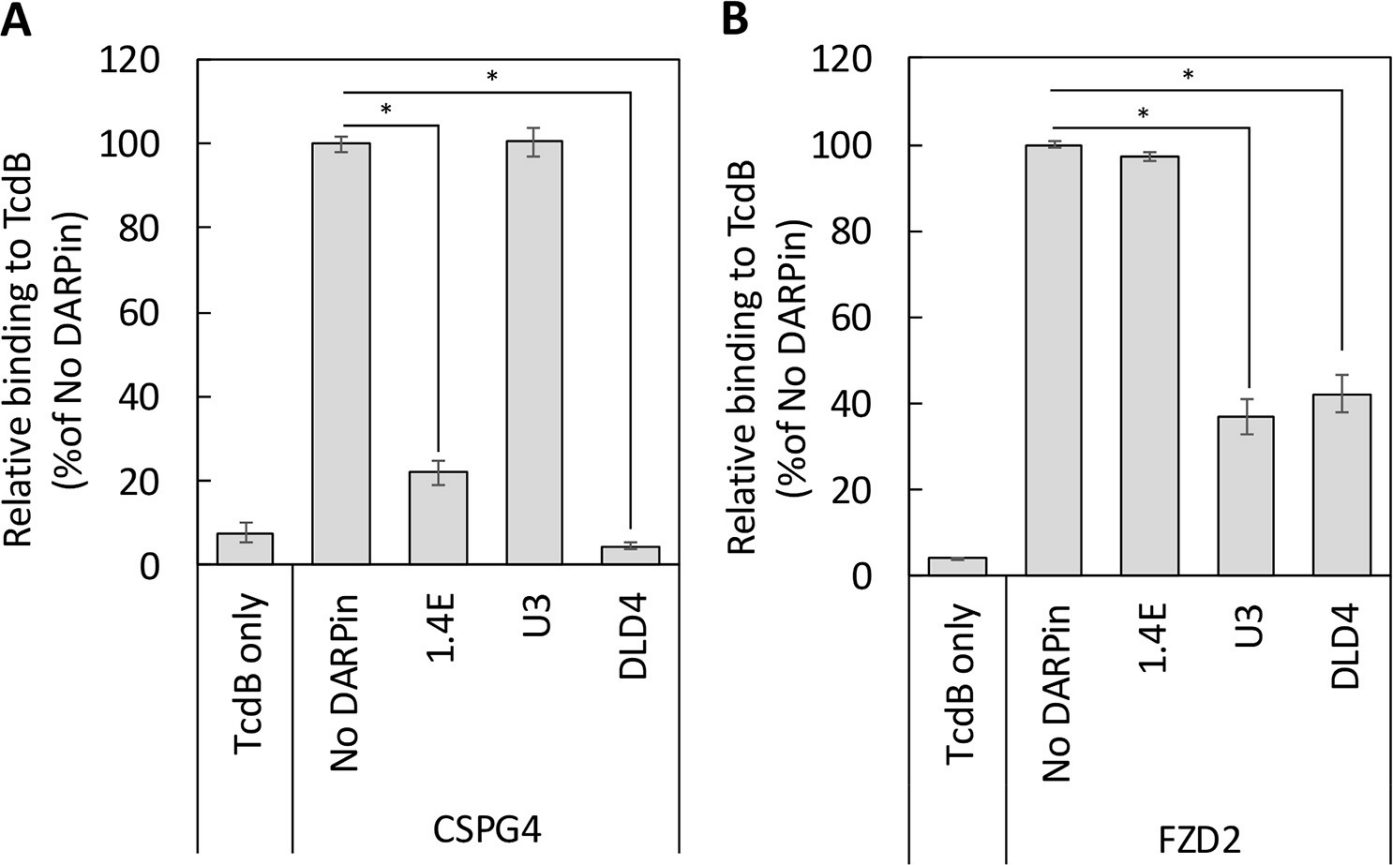
DARPin DLD-4 blocks the interaction between TcdB and its cellular receptors. ELISA plates were coated with TcdB_VPI_ followed by treatment with the 1 nM of the extracellular domains of CSPG4 (A) or FZD2 (B) alone or in mixture with 250 nM of the indicated DARPins. The amount of TcdB-bound CSPG4 and FZD2 was detected using the respective antibodies. Data were normalized to each receptor binding to TcdB in the absence of DARPins (“No DARPin”). Both 1.4E and DLD-4 blocked the interaction between CSPG4 and TcdB, whereas U3 had no significant effect on the TcdB–CSPG4 interaction. Similarly, U3 and DLD-4 significantly blocked the interaction between FZD2 and TcdB but not 1.4E. Error bars represent the standard deviation of 3 independent experiments done in duplicate. *p < 0.005, *t* test. CSPG4, chondroitin sulfate proteoglycan 4; DARPin, designed ankyrin repeat protein; ELISA, enzyme-linked immunosorbent assay; FZD2, frizzled class receptor 2; TcdB, *C*. *difficile* toxin B.

We first determined whether DLD-4 competes with CSPG4 for binding to TcdB using competitive ELISA. A GFP-tagged extracellular domain of CSPG4 (CSPG4-EC-GFP) was expressed in human embryonic kidney (HEK) 293F cells and purified. For the ELISA experiments, MaxiSorp 96-well plates were first coated with purified TcdB and then incubated with CSPG4-EC-GFP (1 nM) in the presence or absence of DLD-4 or its constituent DARPins (250 nM) for 1 hour at room temperature. After thorough washing, the bound CSPG4-EC-GFP was detected using anti-GFP antibody. As shown in Fig 10A, DLD-4 and one of its constituent monomers,1.4E, significantly reduced the binding signal from CSPG4-EC-GFP with DLD-4, producing a more pronounced signal reduction than 1.4E. No significant reduction in binding signal was observed for U3. This result indicates that the monomeric DARPin 1.4E, and by extension the dimeric DARPin DLD-4, interferes with the toxin–CSPG4 interaction.

We carried out a similar competition ELISA experiment to determine whether DLD-4 and FZD2 bind to overlapping epitopes on TcdB. After toxin immobilization, the plate was incubated with the Fc-tagged extracellular domain of FZD2 (FZD2-Fc) (1 nM) in the presence or absence of DLD-4 or its constituent DARPins (250 nM). As shown in Fig 10B, a significant reduction of binding signal was observed for DLD-4 and U3, whereas no reduction of FZD2-binding signal was observed for 1.4E. These data indicate that U3 competes with FZD2 for binding to TcdB. Because U3 interacts with TcdB between residues 1,461 and 1,510 and residues 1,595 and 1,603, our result provided further evidence of the reported binding site of FZD2 on TcdB [48]. No signal reduction was observed for the extracellular domain of PVRL3 (PVRL3-EC) using the same assay (S17 Fig), suggesting that DLD-4 probably does not interfere with the interaction between PVRL3 and TcdB. Taken together, these studies suggest that the high toxin-neutralization potency of DLD-4 in Vero cells likely stems from its ability to simultaneously interfere with the interaction between TcdB and 2 of its cellular receptors, CSPG4 and FZD2.

Because Vero cells express high levels of CSPG4, FZD7, and PVRL3 [58], we sought further confirmation of the toxin-neutralization mechanism of DLD-4 in cells with different receptor expression levels. Caco-2 cells were selected because they are reported to express a high level of frizzled receptor 7 but a negligible level of CSPG4 or PVRL3 [55,58,59]. We anticipate 1.4E, which interferes with TcdB–CSPG4 interaction, to lack any protective activity in these cells, whereas U3, which blocks TcdB–frizzled receptor interaction, to be highly protective. As anticipated, only U3 and DLD-4 showed significant TcdB-neutralization ability in Caco-2 cells, whereas 1.4E showed no protective activity (S18A Fig). The mixture of U3 and 1.4E showed similar efficacy as U3 alone. This result is consistent with our speculation that 1.4E acts by blocking TcdB–CSPG4 interaction. We next tested the ability of these DARPins to neutralize TcdB in a HeLa-derived cell line, TZM, which expresses both CSPG4 and frizzled receptor [55]. Both U3 and 1.4E partially protected HeLa cells against TcdB (S18B Fig). A mixture of these 2 DARPins showed additive activity, whereas DLD-4 exhibited the strongest activity. This result indicates that both of CSPG4 and frizzled receptor mediate TcdB entry in HeLa cells. Finally, because U3 was one of the weakest TcdB inhibitors that emerged from our screening conducted in Vero cells, we decided to evaluate the neutralization ability of all the other monomeric DARPins in Caco-2 cells. Of these, only 8.1B showed significant TcdB-neutralization activity in Caco-2 cells (S19 Fig). This likely reflects an insignificant role of frizzled receptor in Vero cells for TcdB entry.

## Discussion

CDI represents a serious public health problem with more than US$6 billion in annual treatment-associated costs [60]. CDI often occurs post–broad-spectrum antibiotics treatment, which disrupts patients’ natural gut microflora that would normally prevent the colonization of *C*. *difficile*. Consequently, antibiotics are nonideal therapeutics against CDI. In addition, the rate of CDI recurrence post cessation of antibiotic treatment has greatly increased in recent decades [5,6], pointing to a pressing need for nonantibiotic-based CDI therapeutics. Direct toxin neutralization by monoclonal antibodies has emerged as a promising mode of therapy against CDI [61–63], with bezlotoxumab (ZINPLAVA)—a fully human monoclonal antibody against *C*. *difficile* toxin TcdB—recently being approved by the FDA for treatment of recurrent CDI [7,8]. The market value of bezlotoxumab is predicted to reach over US$212 million by 2020 [64]. However, even with bezlotoxumab, the CDI recurrence rate remains high at 15% to 17%[8]. Furthermore, bezlotoxumab shows significantly reduced neutralization potency against toxins from hypervirulent strains of *C*. *difficile* ribotype 027 and 078 [65] (Fig 5).

In this study, we aimed to engineer a highly efficacious microbial-expression compatible antibody surrogate protein, a DARPin, to neutralize *C*. *difficile* toxin TcdB. DARPins represent a versatile class of binding proteins that have been engineered to bind diverse targets with up to picomolar affinity [11–24]. Furthermore, DARPins are also amenable to high-yield production in microbial-expression hosts [10]. Given these attractive properties of DARPins, our long-term goal is to create DARPin-based oral therapeutics against CDI.

Combining phage panning and functional screening, we identified 12 DARPins that protected Vero cells against the TcdB-induced cytopathic effect at nanomolar concentrations (Fig 1). A secondary functional screening of dimeric DARPins yielded 10 dimers with >100-fold improved toxin-neutralization potency relative to the constituent monomers (Fig 2). The best dimer DARPin, DLD-4 (comprising U3 and 1.4E), neutralized TcdB from VPI 10463 (ribotype 087) and M68 (ribotype 017) with EC_50_ values of 4 and 16 pM, respectively, representing an approximately 330-fold and an approximately 33-fold higher potency than the FDA-approved bezlotoxumab in the same assay (Fig 5). DLD-4 effectively protected mice from a lethal IP TcdB toxin challenge (Fig 6A). However, due to the surprisingly poor resistance of DLD-4 against digestion by trypsin and chymotrypsin, no significant protection was observed in the mouse toxin cecum injection model (Fig 6B). Ongoing work in the lab is aimed at improving the protease stability of DLD-4.

Cryo-EM analysis of the complex formed between TcdB and the most potent antitoxin DARPin, DLD-4, revealed that the constituent DARPins of DLD-4, 1.4E and U3, bind to 2 distinct regions of TcdB (Fig 7). Guided by this structural information, we carried out competitive ELISA studies and showed that 1.4E and U3 compete with receptors CSPG4 and FZD2, respectively, for binding to TcdB (Fig 10). No competition between DLD-4 and the third TcdB receptor PVRL3 was observed (S17 Fig). Thus, we conclude that the ultrahigh neutralization potency of DLD-4 mainly derives from its ability to simultaneously interfere with the interaction between TcdB and 2 of its receptors—CSPG4 and FZD2.

Our cryo-EM studies further revealed a novel conformation of TcdB at pH 7.4 in which the CROPS domain points away from the delivery domain. This observation is in contrast to what has been seen in the negative-stain EM structure of TcdA [26,38], as well as the major conformation of the negatively stained apo TcdB (S8 Fig). Unlike negative-stain EM, the cryo-EM specimen preparation is less prone to structural artifacts from specimen preparation. Thus, we believe that our cryo-EM structure likely represents the native conformation of TcdB in an aqueous environment at pH 7.4.

In summary, we report the engineering of a panel of DARPins with superior in vitro toxin-neutralization potency against *C*. *difficile* toxin TcdB than the FDA-approved anti-TcdB monoclonal antibody bezlotoxumab. These highly potent DARPin-based antitoxins possess the potential to be developed into therapeutics to treat CDI and/or prevent its recurrence. Efforts are ongoing to make the molecules from this study amenable to oral administration. Last but not least, cryo-EM structural studies, for the first time, revealed a novel and native conformation of the full-length toxin at pH 7.4 encompassing the CROPS domain, providing important structural insights into the toxin-neutralization mechanism of the most effective antitoxin DARPin in this study, DLD-4. These results should facilitate the design and engineering of new molecules that block the toxic action of TcdB.

## Materials and methods

### Ethics statement

All procedures involving mice were conducted under protocols approved by the Institutional Animal Care and Use Committees at the University of Maryland (IACUC #0517002).

Mice judged to be in a moribund state were euthanized via carbon dioxide asphyxiation.

For the cecum injection experiment, mice were anesthetized with intramuscular injection of a mixture of ketamin (100 mg/kg) and xylazine (10 mg/kg).

### TcdB expression and purification

Plasmid DNA encoding a 6-His–tagged TcdB [66] was transformed into *Bacillus megaterium* cells, and the recombinant TcdB was purified via Ni-NTA affinity column essentially as described previously by Yang and colleagues [66]. The column was washed with high-salt PBS (20 mM NaH_2_PO_4_, 20 mM Na_2_HPO_4_, 300 mM NaCl [pH 7.4]) containing 25 mM imidazole, and the bound protein was eluted using high-salt PBS containing 250 mM imidazole. Eluted protein was diluted in low-salt PBS (20 mM NaH_2_PO_4_, 20 mM Na_2_HPO_4_, 10 mM NaCl [pH 7.4]) to obtain a final NaCl concentration of 30 mM, and the mixture was loaded onto a Q HP anion exchange column (GE Healthcare, Chicago, IL). The column was washed with the same low-salt PBS buffer, and bound protein was eluted using a salt gradient from 10 mM to 1 M NaCl. TcdB eluted at NaCl concentrations of approximately 500 mM. Protein purity was confirmed using SDS-PAGE (S20 Fig).

### DARPin library creation and phage panning

DARPins are composed of repeat modules of natural ankyrin protein and consist of an N-terminal capping repeat (N-cap), 3 (N3C) internal repeats, and a C-terminal capping repeat (C-cap) [9]. In a DARPin library, each internal repeat contains 6 randomized positions, yielding a total of 18 randomized positions in each DARPin. We prepared such a DARPin library using sequential ligation and PCR [16]. This DARPin library was positioned downstream of a DsbAss cotranslational translocation signal peptide [67] and fused to the N-terminus of the bacteriophage M13 gIII minor coat protein. A DARPin library consisting of approximately 2 × 10^9^ unique clones was created by transforming approximately 12 mL high-efficiency MC1061 electro-competent cells with approximately 250 μg of ligated and purified DNA.

Phage panning was carried out as described previously by Steiner and colleagues [68]. TcdB (from *C*. *difficile* VPI10463) was biotinylated via EZ-Link-Sulfo NHS-LC Biotin (Pierce, Waltham, MA) and used as the target protein. Four rounds of sequential phage panning were performed. The enrichment of TcdB-binding DARPin was confirmed by phage ELISA following a published protocol by Steiner and colleagues [68]. A plateau in the level of TcdB binding was observed after round 3 of panning (S21 Fig), indicative of successful phage panning.

To create a dimeric DARPin library, monomeric DARPin variants identified from the functional library screening were PCR-amplified using Taq DNA polymerase (New England BioLabs, Ipswich, MA) with 2 sets of primers (S22 Fig). Set 1 used primers Ran2-D-F and Linker-BSAi-D-R (S2 Table) to generate DARPins with a 3ʹ linker sequence ((GGGGS)x3), and set 2 used primers Linker-BSAi-D-F and Ran2-D-R to generate DARPins with a 5ʹ linker sequence. PCR products were digested with BsaI to generate sticky ends in the added linker region and ligated to form dimeric DARPins. This library was then inserted into the pET28a vector for expression in *E*. *coli* (S22 Fig).

### Functional screening of TcdB-neutralizing DARPins

Pooled DARPin variants from the third round of phage panning were subcloned into the pET26b vector (between NdeI and HindIII) for high-level DARPin expression (S19A Fig). A total of 764 individual clones of *E*. *coli* BL21 (DE3) cells transformed with the enriched library were picked and grown in eight 96-well–deep plates (1 mL/well) at 37°C for 8 to 10 hours. Fifty μL of the overnight culture was transferred to fresh plates containing 1 mL/well LB and grown to mid-log phase (OD_600_ approximately 0.6; approximately 3 hours) prior to the addition of IPTG. The culture was shaken at 400 rpm and at 37°C for 4 hours and was harvested by centrifugation at 1,700*g* for 20 minutes. The cell pellets were resuspended in 100 μL of PBS (1.8 mM KH_2_PO_4_, 10 mM Na_2_HPO_4_, 137 mM NaCl, 2.7 mM KCl [pH 7.4]) supplemented with lysozyme (200 μg/mL), incubated at 37°C for 30 minutes, subjected to 3 cycles of freeze-thaw between 80°C and 37°C, and centrifuged at 16,000*g* for 10 minutes. The soluble fraction was transferred to fresh 96-well–deep plates, incubated at 70°C for 20 minutes and centrifuged again, yielding highly enriched DARPin in the supernatant (S24 Fig). The supernatant was transferred to fresh plates and stored at −80°C until use.

The semipurified DARPin (0.1–10 μL lysate) was incubated with purified TcdB in growth medium (DMEM supplemented with 10% fetal bovine serum [FBS], nonessential amino acids, penicillin [100 mg/mL], and 100 mM streptomycin) in 96-well plates for 2 hours at room temperature and then added to Vero cells seeded the night before in growth medium (final TcdB concentration 132 pg/mL). The concentration of TcdB was selected such that the viability of toxin-treated Vero cells was 10% to 20% that of naïve Vero cells after 6 hours of toxin contact time. Cell supernatants were replaced with fresh growth medium 6 hours later, and the cell viability was quantified 72 hours post toxin addition using CellTiterGlo reagent (Promega) and normalized to Vero cells treated with the equivalent amount of lysate from untransformed BL21(DE3) cells in the absence of TcdB.

For the dimeric DARPin functional screen, the protocol was further simplified. A total of 1,504 individual clones of *E*. *coli* BL21 (DE3) cells transformed with the dimeric DARPin library were picked and grown in 16 deep 96-well plates (1 mL/well) at 37°C and 400 rpm in LB overnight. The next day, the cultures were harvested by centrifugation at 1,700*g* for 20 minutes. Each of the cell pellets was resuspended in 200 μL PBS supplemented with lysozyme (200 μg/mL) and incubated at 37°C for 30 minutes. Next, the plates were subjected to 1 cycle of freeze-thaw between −80°C and 37°C and incubated at 70°C for 20 minutes. The lysate was diluted in PBS, and an equivalent of 0.2 μL of the undiluted lysate was added to Vero cells together with TcdB toxin (10 pg/mL) in a final volume of 100 μL; 72 hours later, the cell viability was quantified by CellTiterGlo assay and normalized to that of naïve Vero cells.

### Protein expression and purification

*E*. *coli* BL21 (DE3) cells transformed with DARPin expression plasmid (in pET26b, S3 Fig) were cultured overnight at 37°C in autoinduction media (6 g/L Na_2_HPO_4_, 3 g/L KH_2_PO_4_, 20 g/L tryptone, 5 g/L yeast extract, 5 g/L NaCl, 0.6% glycerol, 0.1% glucose, 0.08% lactose) supplemented with 50 μg/mL kanamycin. Cells were lysed by sonication. The lysate was clarified by centrifugation at 16,000*g* for 10 minutes, and the soluble lysate was filtered through a 0.45 μm PES membrane and loaded onto a gravity Ni-NTA agarose column. The column was washed with PBS containing 15 mM imidazole, and the bound proteins were eluted using PBS containing 150 mM imidazole. Protein purity was determined using SDS-PAGE (S25 Fig).

DNA encoding bezlotoxumab VH and VL were synthesized, and construct-encoding bezlotoxumab IgG1 light and heavy chains were transfected to CHO cells. The bezlotoxumab was purified from CHO supernatants using protein-A beads following a standard protocol.

The gene of the CSPG4 extracellular domain (410–560) and its signal peptide was synthesized and inserted into the pEGFP-N1 plasmid upstream of the GFP gene between the restriction sites NdeI and BamHI. The expression plasmid was used to transfect 293 F cells, and the expressed protein was secreted into the medium. To purify the expressed CSPG4-EC-GFP, streptavidin beads (Novagen) were first coated with anti-GFP nanobody fused to an AviTag purified from BirA+ *E*.*coli* [69]. After thorough washing with tris buffer (50 mM tris, 150 mM NaCl [pH 7.5]), the beads were incubated with medium containing the secreted CSPG4-EC-GFP overnight at 4°C. The beads were next washed with tris buffer, and the CSPG4-EC-GFP protein was released into supernatant via sumo protease digestion, which separates the AviTag from the fusion protein.

The FBD domain of TcdB_VPI_ (amino acid 1,285–1,804) was cloned into pET28a (harboring an N-terminal 6× His tag) and purified on a Ni-NTA agarose column [48]. The final purified protein was concentrated and buffer exchanged into PBS by ultrafiltration (50 kDa MWCO).

### ELISA

MaxiSorp immunoplates (Nunc) were coated with 4 μg/mL TcdB overnight at 4°C. The next day, the wells were washed and blocked with PBSTB buffer (PBS containing 0.1% Tween-20 and 2% BSA) before being incubated with serially diluted IMAC-purified DARPins (containing an myc-tag at the N-terminus; S3 Fig), CSPG4-EC-GFP, FZD2-EC (R&D Systems, Minneapolis, MN), or PVRL3 (Sino Biological, Wayne, PA). After incubation, wells were washed 4 times with PBST. Bound DARPins were detected using mouse anti-c-myc antibody (1 μg/mL, Invitrogen [catalog #13–2500]) and HRP-conjugated anti-mouse antibody (0.8 μg/mL, Jackson ImmunoResearch, West Grove, PA [catalog #115-035-146]). Bound CSPG4-EC-GFP was detected using rabbit anti-GFP antibody (0.05 μg/mL, Proteintech, Rosemont, IL [catalog #50430-2-AP]) and HRP-conjugated anti-rabbit antibody (0.8 μg/mL, Santa Cruz Biotechnology, Dallas, TX [catalog #SC-2004)]. HRP-conjugated goat anti-human antibody (0.025 μg/mL, JacksonImmunoResearch, West Grove, PA [catalog #109-035-088]) was used to detect bound FZD2-EC. The color development agent was 3,3',5,5'-tetramethylbenzidine (TMB).

### In vitro TcdB-neutralization assay

Vero cells (1.5 × 10^3^ cells/well) in growth medium were seeded in 96-well plates. The next day, serial dilutions of IMAC-purified DARPins were added to the appropriate wells followed by the addition of TcdB (final concentration 5 pg/mL or 2.5 pg/mL). The concentration of TcdB was selected such that the viability of the toxin-treated cells was 10% to 20% that of naïve Vero cells following 72 hours of toxin contact time. The plates were incubated at 37°C /5% CO_2_ for 72 hours, and the cell viability was quantified using the CellTiterGlo kit (Promega, Madison, WI).

### In vivo TcdB-neutralization activity of DARPins

Six- to eight-week-old CD1 mice were purchased from Harlan Laboratories (Indianapolis, IN). All mice were housed in dedicated pathogen-free facilities in groups of 5 mice per cage under the same conditions. Food, water, bedding, and cages were autoclaved. All procedures involving mice were conducted under protocols approved by the Institutional Animal Care and Use Committees at the University of Maryland (IACUC #0517002). Mice judged to be in a moribund state were euthanized via carbon dioxide asphyxiation. DLD-4 (2.5 mg/kg or 0.25 mg/kg) or bezlotoxumab (10 mg/kg) was mixed with TcdB (1.5 **μ**g/kg) in PBS and incubated at room temperature for 1 hour before being injected IP into mice in the appropriate treatment groups. The control group was IP injected with TcdB alone in PBS. Mouse survival was monitored for 4 days until the termination of the experiments, and data were analyzed by Kaplan–Meier survival analysis with log-rank test of significance. The cecum injection experiment was carried out as described previously by Zhang and colleagues [35]. Mice were anesthetized with intramuscular injection of a mixture of ketamine (100 mg/kg) and xylazine (10 mg/kg). The cecum, ileum, and colon were exposed upon a midline laparotomy. IMAC-purified TcdB_VPI_ (15 **μ**g/mouse) or a mixture of TcdB_VPI_ and DLD-4 (5 mg/mouse) in PBS (100 **μ**L) was injected directly into the cecum of mice via insulin syringes (29G) inserted into the ileocecal junction. The gut was returned to the abdomen after injection, and the incision was closed with silk sutures. Mice were allowed to recover, and mouse survival was closely monitored for 72 hours.

### EM sample preparation

TcdB and DLD-4 were mixed at 1:1 molar ratio (with the final concentration of the complex at 800 nM) and incubated in PBS buffer at pH 7.4 for 30 minutes at room temperature; 3 μL of the complex was applied to C-flat 1.2/1.3 holey carbon film 400 mesh grids at 20°C with 100% relative humidity and vitrified using a Vitrobot (Mark III, FEI Company, the Netherlands). Apo-state TcdB was prepared in the same buffer and vitrified under the same conditions but without the incubation at room temperature.

### Cryo-EM data collection

The complex of TcdB and DLD-4 was imaged under both the FEI Tecnai F20 electron microscope (FEI Company, the Netherlands) and a JEOL JEM3200FSC electron microscope (JEOL, Japan), with their respective field emission guns operated at 200 kV and 300 kV. Each microscope is equipped with a Gatan K2 summit direct detection camera (Gatan, Pleasanton, CA); 2,255 (from Tecnai F20) and 1,647 (from JEM3200FSC) micrographs were collected using macro mode or manual mode of SerialEM [70] in the super-resolution electron-counting mode. Nominal magnifications of 25,000X (on Tecnai F20) and 30,000X (on JEM3200FSC) were used, yielding subpixel sizes of 0.75 Å and 0.615 Å, respectively. The beam intensity was adjusted to 5 e^−^/Å^2^/s on the camera. A 33-frame movie stack was collected for each picture, with 0.2 seconds per frame, for a total exposure time of 6.6 seconds. For data collected on the JEM3200FSC, an in-column energy filter was used with a slit width of 29 eV.

As a control, the apo-state TcdB was imaged similarly but under the FEI Tecnai F20 electron microscope only. Image data were collected under nominal magnifications of both 25,000X (200 micrographs) and 29,000X (200 micrographs), yielding subpixel sizes of 0.75 Å and 0.62 Å, respectively.

### Image processing

Movie stacks, collected on both microscopes for the complex of TcdB and DLD-4, were first binned by 2 to yield pixel sizes of 1.5 Å and 1.23 Å. Using Unblur [71], these stacks were aligned and summed to generate a set from frames 1–33. These sum images were visually screened, and 1,272 and 1,173 of each set with strong power spectra were selected for further processing. Contrast transfer functions of the micrographs were estimated using CTFFind4 [72]. Batchboxer in EMAN [73] was first used to automatically pick all the particles from 1,272 sum images with a box size of 216 pixels^2^, or particles from 1,173 sum images with a box size of 280 pixels^2^, yielding 331,516 particles and 234,642 particles, respectively. These particles were scaled to a common pixel size of 3 Å by scaling the extracted particles using Relion [73]. The automatically picked particles were then screened for high-contrast particles for 4 rounds of the reference-free 2D classification in Relion [74]; 45,806 and 38,614 clean particles were selected and combined for 3D classification, separating particles into 4 classes. Because density maps from these classes had similar conformations except for the CROPS domain, all the clean particles were used for 3D refinement to generate a final density map of TcdB–DLD-4 complex at 9 Å resolution, which is better than the maps reconstructed from the individual data set from each microscope (S5 Fig). The same particles were also used for 3D refinement of the core region with a solvent mask that masked out the CROPS region, generating a density map of the TcdB–core–DLD-4 complex at 8.3 Å resolution (S4 Fig). The overall resolution was assessed using the gold-standard criterion of Fourier Shell Correlation [75], with a cutoff at 0.143, between 2 half maps from 2 independent half-sets of data. Local resolutions were estimated using Blocres [76]. The apo TcdB structure was similarly processed.

The image data for the apo-state TcdB were processed similarly to a resolution of 24 Å from a total of 27,584 particles.

### Model building

We used the higher-resolution density map of the DLD-4-bound TcdB for the model building. Briefly, the homology models for the core region of the TcdB (residues 1–1,799) and the U3 and 1.4E modules of the DLD-4, were built in Swiss-Model [77]. The linker between U3 and 1.4E was manually built in UCSF Chimera [78]. These models, along with the crystal structure of the N-terminal half of the CORPS domain in TcdB (residues 1,834–2,101, PDB code: 4NP4) were combined in UCSF Chimera [78] and refined into the cryo-EM density map using Molecular Dynamics Flexible Fitting [79] to generate the complex structure of TcdB and DLD-4. I-TASSER [80] was used to predict the secondary structure for the region of residues 1,800–1,834.

## Supporting information

S1 Fig Sandwich ELISA to evaluate the ability of a dimer DARPin to bind to 2 different TcdB molecules. ELISA plates were coated with TcdB (4 μg/mL, overnight at 4°C) followed by treatment with 250 nM of the indicated DARPin constructs. After thorough washing, biotinylated TcdB was added to the wells. Biotinylated TcdB captured by DARPins was detected using HRP-conjugated streptavidin. Only the construct linking DARPins binding to an overlapping epitope (i.e., 1.4E-L-1.2E) was able to bind to both immobilized and solution phase TcdB molecules simultaneously. Data are representative of 3 independent experiments. DARPin, designed ankyrin repeat protein; ELISA, enzyme-linked immunosorbent assay; HRP, horseradish peroxidase; TcdB, *C*. *difficile* toxin B.
(TIF)

S2 Fig DARPin dimers offer superior inhibition of TcdB from *C*. *difficile* strain VPI 10463 (ribotype 087). IMAC-purified DARPin dimer DLD-4 or bezlotoxumab were added to (1.5 × 10^3^ cells/well) Vero cells (1.5 × 10^3^ cells/well) together with TcdB (5 pg/mL). Cell viability was quantified 72 hours later using a cell rounding assay (A) or the CellTiterGlo assay (B) and normalized to naïve Vero cells. (C) TcdB-neutralization potency. Error bars represent the standard deviation of duplicate wells. Data presented are representative of 2 independent experiments. To quantify cell rounding, phase-contrast images were taken with an Olympus microscope. The numbers of normal and rounded cells in each image were determined by counting manually. DARPin, designed ankyrin repeat protein; IMAC, Immobilized metal affinity chromatography; TcdB, *C*. *difficile*toxin B.
(TIF)

S3 Fig Activities of monomeric DARPins toward UK1 TcdB. (A) Monomeric DARPins show reduced activity against TcdB_UK1_ in Vero cells at nanomolar concentrations. IMAC-purified DARPins were added to Vero cells (1.5 × 10^3^ cells/well) together with TcdB toxin (5 pg/mL). Cell viability was quantified 72 hours later by the CellTiterGlo assay and normalized to naïve Vero cells. Error bars represent the standard deviation of 2 independent experiments done in duplicate for samples showing anti-TcdB activity. (B) Relative binding of selected DARPins to UK1 TcdB was determined using ELISA. Serially diluted DARPins were added to microtiter plates coated with 4 μg/mL of TcdB. Results are representative of 2 independent experiments. DARPin, designed ankyrin repeat protein; ELISA, enzyme-linked immunosorbent assay; IMAC, Immobilized metal affinity chromatography; TcdB, *C*. *difficile* toxin B.
(TIF)

S4 Fig Activities of dimeric DARPins toward UK1 TcdB. (A) Dimeric DARPins show reduced activity against UK1 TcdB in Vero cells at nanomolar concentrations. IMAC-purified DARPins were added to Vero cells (1.5 × 10^3^ cells/well) together with TcdB (5 pg/mL). Cell viability was quantified 72 hours later by the CellTiterGlo assay and normalized to naïve Vero cells. Error bars represent the standard deviation of triplicate samples. (B) Relative binding of selected dimeric DARPins to UK1 TcdB was determined using ELISA. Serially diluted DARPins were added to microtiter plates coated with 4 μg/mL of TcdB. Results are representative of 2 independent experiments. DARPin, designed ankyrin repeat protein; ELISA, enzyme-linked immunosorbent assay; IMAC, Immobilized metal affinity chromatography; TcdB, *C*. *difficile* toxin B.
(TIF)

S5 Fig Bezlotoxumab failed to protect mice from IP-injected TcdB_VPI_. IP, intraperitoneally; TcdB, *C*. *difficile* toxin B.
(TIF)

S6 Fig DLD-4 is sensitive to protease digestion. IMAC-purified DARPins were incubated with 1 mg/ml trypsin or chymotrypsin in PBS for 1 hour before being diluted in complete growth medium and added to Vero cells together with TcdB (5 pg/mL). Cell viability was quantified 72 hours later by the CellTiterGlo assay and normalized to naïve Vero cells. Error bars represent the standard deviation of 2 independent experiments done in duplicate. DARPin, designed ankyrin repeat protein;; IMAC, Immobilized metal affinity chromatography; TcdB, *C*. *difficile* toxin B.
(TIF)

S7 Fig Micrograph, 2D class averages, FSC, and local resolutions of DLD-4-bound TcdB. FSC, Fourier Shell Correlation; TcdB, *C*. *difficile* toxin B.
(TIF)

S8 Fig Data processing procedure for the DLD-4-bound TcdB and its flexibility at the tip of the CROPS domain. CROPS, combined repetitive oligopeptides; TcdB, *C*. *difficile* toxin B.
(TIF)

S9 Fig 2D class averages of the apo TcdB at pH 7.4 generated through negative-stain EM. Yellow arrows label the tip of the delivery domain, and green arrows label the CROPS domain. In 80% of the negatively stained particles, the CROPS domain extends toward the delivery domain. This illustrates the problem for the negative-staining EM with this specimen. Only 20% of the data are in a similar conformation as observed in cryo-EM. Purified full-length TcdB (VPI 10463, 0.01 mg/mL) was applied on glow-discharged 400 mesh carbon-coated grids. The sample was stained by immersing in 0.75% uranyl acetate (w/v) for 30 seconds. The prepared grid was loaded and imaged under an FEI Tecnai F20 electron microscope with a field emission gun (FEI Company, the Netherlands) operated at 200 kV, yielding 60 micrographs. Data were collected on a Gatan K2 summit direct detection camera (Gatan, Pleasanton, CA) in the electron-counting mode. A nominal magnification of 19,000 X was used, yielding a pixel size of 1.87 Å. CROPS, combined repetitive oligopeptides; cryo-EM, cryo-electron microscopy; EM, electron microscopy; TcdB, *C*. *difficile* toxin B.
(TIF)

S10 Fig Cryo-EM maps of DLD-4-bound TcdB. (A) Fitting the cryo-EM map of the DLD-4-bound TcdB (magenta) into cryo-EM map of the apo TcdB (gray transparent) to show the structural similarity between the 2 states. The delivery domain and CROPS domain are indicated. In both states, the CROPS domain protrudes away from the delivery domain. (B) 3D classification of apo TcdB conformations. Side views (top panel) and bottom views (bottom panel) are shown. The orientations of the 4 classes in the side views are the same as in panel A. The number under each class number indicates the percentage of the total number of particles generating that class. CROPS, combined repetitive oligopeptides; cryo-EM, cryo-electron microscopy; TcdB, *C*. *difficile* toxin B.
(TIF)

S11 Fig Comparison of the structure of apo TcdA and apo TcdB. PDB structure of TcdA (4R04) is colored in yellow, and the model of TcdB is colored in blue. Panel B is the view obtained by turning 90 degrees along the horizontal line relative to the view in panel A. Panel C is the view obtained by turning 90 degrees along the horizontal line relative to the view in panel B. PDB, protein data bank; TcdA, *C*. *difficile* toxin A; TcdB, *C*. *difficile* toxin B.
(TIF)

S12 Fig Comparison of TcdB from VPI 10463 and UK1. (A) Sequence differences between the TcdB from VPI 10463 and UK1 are labeled gray and orange, with the orange color labeling the residues at the interface of the TcdB and the DARPin. The predicted secondary structure is shown below the sequence. (B) Two views of the β-sheet–like density (within the dashed oval), belonging to residues 1,801 to 1,839, which are currently not modeled. DARPin, designed ankyrin repeat protein; TcdB, *C*. *difficile* toxin B.
(TIF)

S13 Fig **ELISA data of DARPin 1.4E (A) and U3 (B) mutants binding to TcdB from the VPI strain of *C*. *difficile*.** IMAC-purified DARPins were serially diluted and added to microtiter plates coated with 4 μg/mL of TcdB. ELISA experiment was carried out as described in the Materials and methods section. Data obtained were normalized to values obtained for the unmodified 1.4E and U3. Error bars represent the standard deviation of 2 independent experiments. (C) DARPin 1.4E mutants show similar activity, whereas U3 mutants show reduced activity against VPI TcdB in Vero cells. IMAC-purified DARPins were added to Vero cells (1.5 × 10^3^ cells/well) together with TcdB toxin (5 pg/mL). Cell viability was quantified 72 hours later by the CellTiterGlo assay and normalized to naïve Vero cells. Error bars represent the standard deviation of 2 independent experiments done in duplicate. DARPin, designed ankyrin repeat protein; ELISA, enzyme-linked immunosorbent assay; IMAC, immobilized metal ion affinity chromatography; TcdB, *C*. *difficile* toxin B.
(TIF)

S14 Fig **ELISA data of DARPins (A) U3 and (B) 1.4E binding to TcdB from different strains of *C*. *difficile*.** IMAC-purified DARPins were serially diluted and added to microtiter plates coated with 4 μg/mL of TcdB. ELISA experiment was carried out as described in the Materials and methods section. DARPin, designed ankyrin repeat protein; ELISA, enzyme-linked immunosorbent assay; IMAC, immobilized metal ion affinity chromatography; TcdB, *C*. *difficile* toxin B.
(TIF)

S15 Fig DARPin 1.4E does not significantly impair the IP6 TcdB autocleavage. TcdB_VPI_ (20 μM) in tris buffer (20 mM [pH 8]) was incubated with 20 μM IP6 in the absence or presence of the different DARPins at the indicated concentration at 37°C for 1 hour and analyzed on 4% to 20% Mini-PROTEAN TGX gels. (1) Full-length TcdB_VPI_; (2) C-terminal delivery and CROPS domains; (3) N-terminal GTD domain. iD: an irrelevant DARPin used here as negative control. It appears that a low level of autocleavage inhibition is present at high protein concentration regardless of the identity of the protein. CROPS, combined repetitive oligopeptides; DARPin, designed ankyrin repeat protein; GTD, glucosyltransferase domain; IP6, inositol hexaphosphate; TcdB, *C*. *difficile* toxin B.
(TIF)

S16 Fig TcdB cryo-EM structural overlay. Overlay of the cryo-EM TcdB structure (pink) generated in this work with the TcdB binding domain crystal structure (yellow, PDB 6C0B). Structural alignment indicates complete overlap between FZD2 (green) and U3 (light purple). cryo-EM, cryo-electron microscopy; FZD2, frizzled class receptor 2; PDB, protein data bank; TcdB, *C*. *difficile* toxin B.
(TIF)

S17 Fig DARPin DLD-4 does not block the interaction between TcdB and PVRL3-EC. ELISA plates were coated with TcdB followed by treatment with PVRL3-EC alone or mixtures of PVRL3-EC with 250 nM DLD-4. The presence of DLD-4 did not affect the binding of PVRL3-EC to TcdB. Error bars depict the standard deviation of 3 independent experiments. DARPin, designed ankyrin repeat protein; PVRL3-EC, extracellular domain of PVRL3; TcdB, *C*. *difficile* toxin B.
(TIF)

S18 Fig DARPin DLD-4 inhibits TcdB from *C*. *difficile* strains VPI 10463 (ribotype 087) in Caco-2 and TZM cells. (A) IMAC-purified DARPins were added to Caco-2 cells (1.5 × 10^3^ cells/well) together with 10 pg/mL TcdB. Cell viability was quantified 72 hours later by the CellTiterGlo assay and normalized to naïve Caco-2 cells. Only U3 and DLD-4 inhibited TcdB cytotoxicity in these cells. Error bars represent the standard deviation of 2 independent experiments done in duplicate. (B) IMAC-purified DARPins were added to TZM cells (1.5 × 10^3^ cells/well) together with 5 pg/mL TcdB. Cell viability was quantified 72 hours later by the CellTiterGlo assay and normalized to naïve TZM cells. U3 and 1.4E showed partial inhibition of TcdB in TZM cells. Error bars represent the standard deviation of 2 independent experiments done in duplicate. DARPin, designed ankyrin repeat protein; IMAC, immobilized metal ion affinity chromatography; TcdB, *C*. *difficile* toxin B.
(TIF)

S19 Fig Most monomeric DARPins do not protect Caco-2 cells from VPI TcdB cytopathy. IMAC-purified DARPins were added to Caco-2 cells (1.5 × 10^3^ cells/well) together with TcdB toxin (5 pg/mL). Cell viability was quantified 72 hours later by the CellTiterGlo assay and normalized to naïve Vero cells. Data are representative of 2 independent experiments done in duplicate for DARPins showing anti-TcdB activity. DARPin, designed ankyrin repeat protein; IMAC, immobilized metal ion affinity chromatography; TcdB, *C*. *difficile* toxin B.
(TIF)

S20 Fig Purification of recombinant TcdB. TcdB was expressed and purified as indicated in the Materials and methods section. Purified TcdB (lane 2) was resolved in a 12% poly-acrylamide gel using SDS-PAGE. The gel was stained with Coomassie blue to visualize TcdB. TcdB, *C*. *difficile* toxin B.
(TIF)

S21 Fig Enrichment of TcdB-binding DARPins after phage panning. A MaxiSorp 96-well plate was coated with neutravidin (0.2 mg/mL) and then biotin-TcdB. The binding of phage recovered from successive rounds of panning to TcdB was quantified using ELISA. Significant binding was observed after 2 rounds of panning. Binding appeared to plateau after the third round of panning. DARPin, designed ankyrin repeat protein; ELISA, enzyme-linked immunosorbent assay; TcdB, *C*. *difficile* toxin B.
(TIF)

S22 Fig DARPin dimer library assembly. Individual DARPins were amplified using indicated primer sets. Primer Linker-BsaI-D-R added the linker sequence and the type 2 restriction endonuclease BsaI to the 3ʹ end of each DARPin. Primer Linker-BsaI-D-F added the linker sequence and the type 2 restriction endonuclease BsaI to the 5ʹ end of each DARPin. DARPin dimers were then assembled the linker by using a golden gate approach by digestion with BsaI, followed by ligation with T4 DNA ligase. DARPin, designed ankyrin repeat protein.
(TIF)

S23 Fig DARPin sequences and schematic. In each DARPin construct there is an N-terminal hexa-His tag followed by an myc tag (A). In the dimeric construct DARPins are separated by a (GGGS)x3 linker sequence (B). DARPin, designed ankyrin repeat protein.
(TIF)

S24 Fig Crude purification of monomeric DARPins. *E*. *coli* BL21(DE3) cells transformed with a monomeric DARPin were grown in a well of a 96-well–deep plates (1 mL/well) at 37°C for 8 to 10 hours. Fifty μL of the overnight culture was transferred to fresh plates containing 1 mL/well LB and grown until OD600 approximately 0.6 (approximately 3 hours) prior to the addition of IPTG. The culture was shaken at 400 rpm and at 37°C for 4 hours and was harvested by centrifugation at 1,700*g* for 20 minutes. The cell pellets were resuspended in 100 μL of PBS supplemented with lysozyme (200 μg/mL), incubated at 37°C for 30 minutes, subjected to 3 cycles of freeze-thaw between −80°C and 37°C and centrifuged at 16,000*g* for 10 minutes. The soluble fraction was transferred to fresh 96-well deep plates and incubated at 70°C for 20 minutes and centrifuged again, yielding highly enriched DARPin in the supernatant. The red arrow indicates DARPin. DARPin, designed ankyrin repeat protein; P, Pellet; S, Soluble fraction.
(TIF)

S25 Fig Purification of DARPins. DARPins were expressed and purified as indicated in the Materials and methods section. Selected DARPin dimers (lanes 2–5) and DARPin monomer (lane 6) were resolved in a 12% poly-acrylamide gel using SDS-PAGE. The gel was stained with Coomassie blue to visualize the purified protein. DARPIN, designed ankyrin repeat protein.
(TIF)

S1 Table DARPin sequences. DARPIN, designed ankyrin repeat protein.
(TIF)

S2 Table Primers used to construct DARPin dimer library. Nucleotides binding to individual DARPins are indicated in lowercase. Nucleotides encoding BsaI restriction sites are underlined. Nucleotides encoding the linker sequence are indicated in bolded letters. Primer Ran2-D-F bound to the 5ʹ end of each DARPin. Primer Ran2-D-R binds to the 3ʹ end of each DARPin. Primer Linker-BSAi-D-F binds to the 5ʹ end of each DARPin, adding a linker sequence and the BsaI restriction site to that end. Primer Linker-BSAi-D-R binds to the 3ʹ end of each DARPin, adding a linker sequence and the BsaI restriction site to that end. As such, primer pairs Ran2-D-F and Linker-BsaI-R were used to amplify a single DARPin, adding the linker and the BsaI site to the 3ʹ end. Similarly, primer pairs Ran2-D-R and Linker-BsaI-F were used to amplify a single DARPin, adding the linker and the BsaI site to the 5ʹ end. DARPIN, designed ankyrin repeat protein.
(TIF)

S1 Data Relevant data values. (XLSX)

S1 Movie Overlay of the crystal structure of apo TcdA and the cryo-EM structure of TcdB. cryo-EM, cryo-electron microscopy; TcdA, *C*. *difficile* toxin A; TcdB, *C*. *difficile* toxin B.
(M4V)

## Abbreviations

APD: autoprocessing domain
AR: ankyrin repeat
C-cap: C-terminal capping repeat
Cdc42: cell division cycle 42
CDI: *Clostridium difficile* infection
CROPS: combined repetitive oligopeptides
cryo-EM: cryo-electron microscopy
CSPG4: chondroitin sulfate proteoglycan 4
CSPG4-EC-GFP: GFP-tagged extracellular domain of CSPG4
CTG: CellTiter-Glo
DARPin: designed ankyrin repeat protein
EC_50_: half maximal effective concentration
ELISA: enzyme-linked immunosorbent assay
EM: electron microscopy
FBD: frizzled binding domain
FBS: fetal bovine serum
FDA: Food and Drug Administration
FZD2: frizzled class receptor 2
FZD2-Fc: Fc-tagged extracellular domain of FZD2
GTD: glucosyltransferase domain
HEK: human embryonic kidney
IP: intraperitoneally
IP6: inositol hexaphospha
N-cap: N-terminal capping repeat
PVRL3: poliovirus receptor like 3
PVRL3-EC: extracellular domain of PVRL3
Rac1: Rac family small GTPase 1
RhoA: Ras homolog gene family, member A
TcdA: *C*. *difficile* toxin A
TcdB: *C*. *difficile* toxin B
TMB: tetramethylbenzidine
