# Correction: Selection and characterization of ultrahigh potency designed ankyrin repeat protein inhibitors of *C. difficile* toxin B

**DOI:** 10.1371/journal.pbio.3000514

**Published:** 2019-10-04

**Authors:** Rudo Simeon, Mengqiu Jiang, Ana M. Chamoun-Emanuelli, Hua Yu, Yongrong Zhang, Ran Meng, Zeyu Peng, Joanita Jakana, Junjie Zhang, Hanping Feng, Zhilei Chen

## Notice of Republication

This article was republished on September 17, 2019, as S14-S19 Figs were attributed to the wrong figure numbers. Please download this article again to view the correct images, figure numbers, and legends.
