# Supplementary figures and images for: Selection and characterization of ultrahigh potency designed ankyrin repeat protein inhibitors of C. difficile toxin B
Source: PLoS Biol. 2019 Jun 24;17(6):e3000311. doi: 10.1371/journal.pbio.3000311 (PMC6590788; doi:10.1371/journal.pbio.3000311)

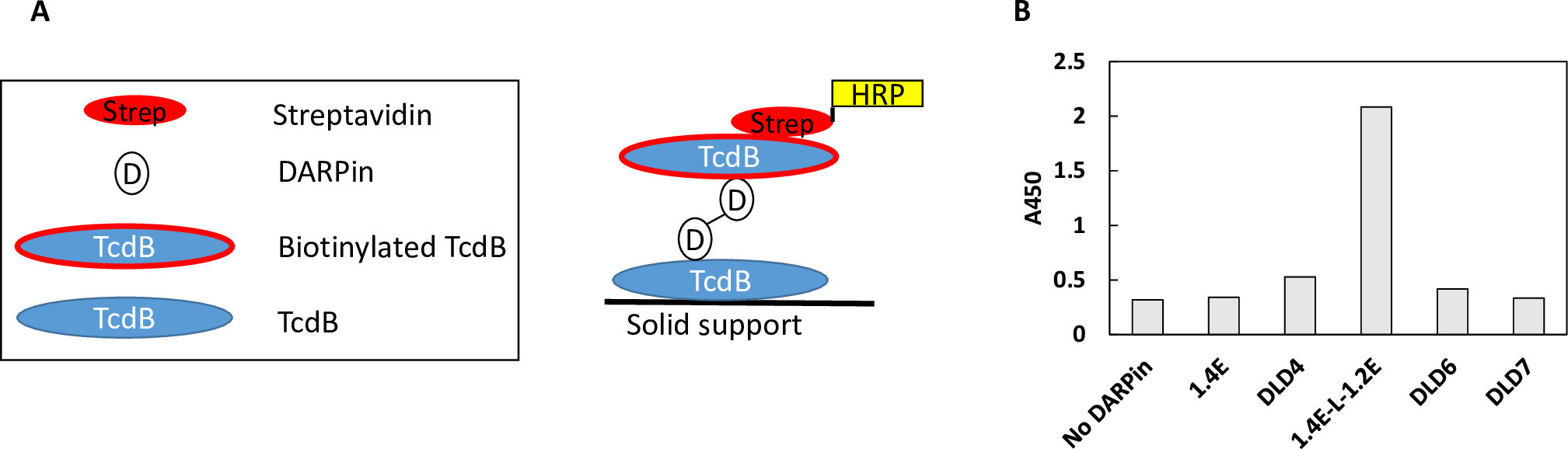

Supplement: S1 Fig — ELISA plates were coated with TcdB (4 μg/mL, overnight at 4°C) followed by treatment with 250 nM of the indicated DARPin constructs. After thorough washing, biotinylated TcdB was added to the wells. Biotinylated TcdB captured by DARPins was detected using HRP-conjugated streptavidin. Only the construct linking DARPins binding to an overlapping epitope (i.e., 1.4E-L-1.2E) was able to bind to both immobilized and solution phase TcdB molecules simultaneously. Data are representative of 3 independent experiments. DARPin, designed ankyrin repeat protein; ELISA, enzyme-linked immunosorbent assay; HRP, horseradish peroxidase; TcdB, C. difficile toxin B. (TIF) [file pbio.3000311.s001.tif]

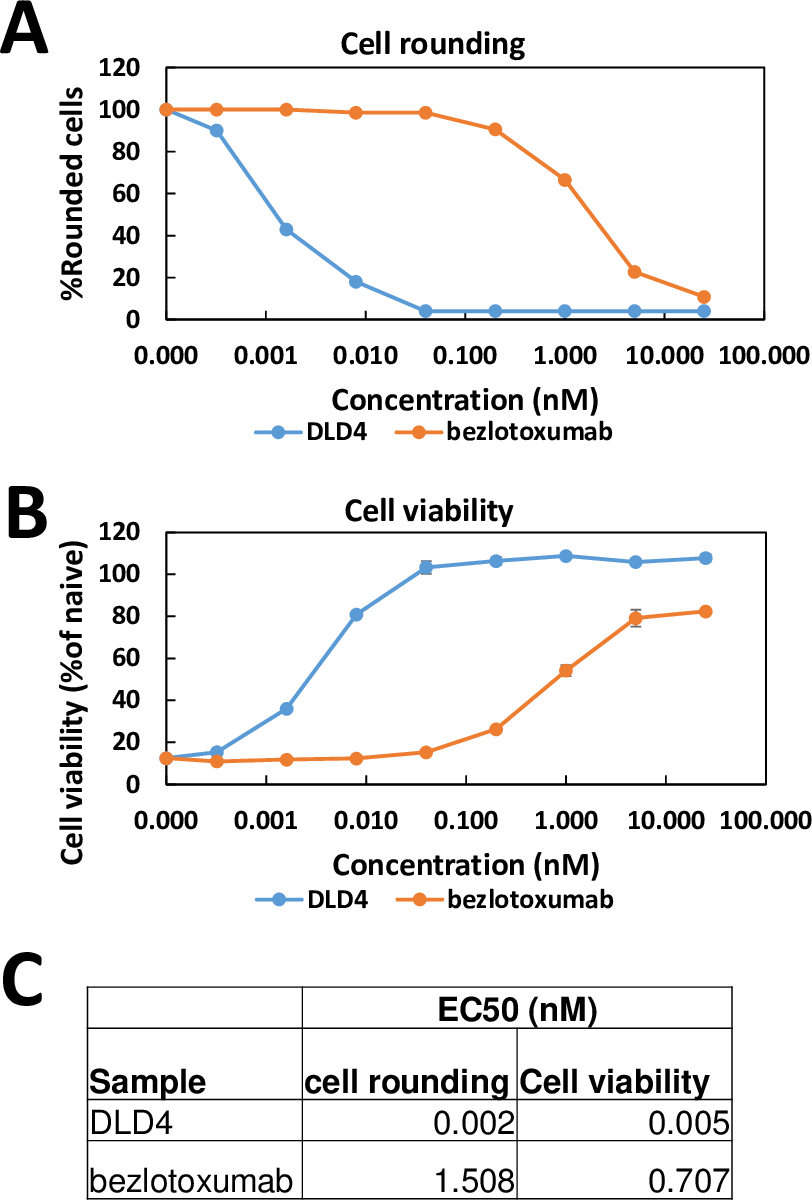

Supplement: S2 Fig — IMAC-purified DARPin dimer DLD-4 or bezlotoxumab were added to (1.5 × 10^3^ cells/well) Vero cells (1.5 × 10^3^ cells/well) together with TcdB (5 pg/mL). Cell viability was quantified 72 hours later using a cell rounding assay (A) or the CellTiterGlo assay (B) and normalized to naïve Vero cells. (C) TcdB-neutralization potency. Error bars represent the standard deviation of duplicate wells. Data presented are representative of 2 independent experiments. To quantify cell rounding, phase-contrast images were taken with an Olympus microscope. The numbers of normal and rounded cells in each image were determined by counting manually. DARPin, designed ankyrin repeat protein; IMAC, Immobilized metal affinity chromatography; TcdB, C. difficiletoxin B. (TIF) [file pbio.3000311.s002.tif]

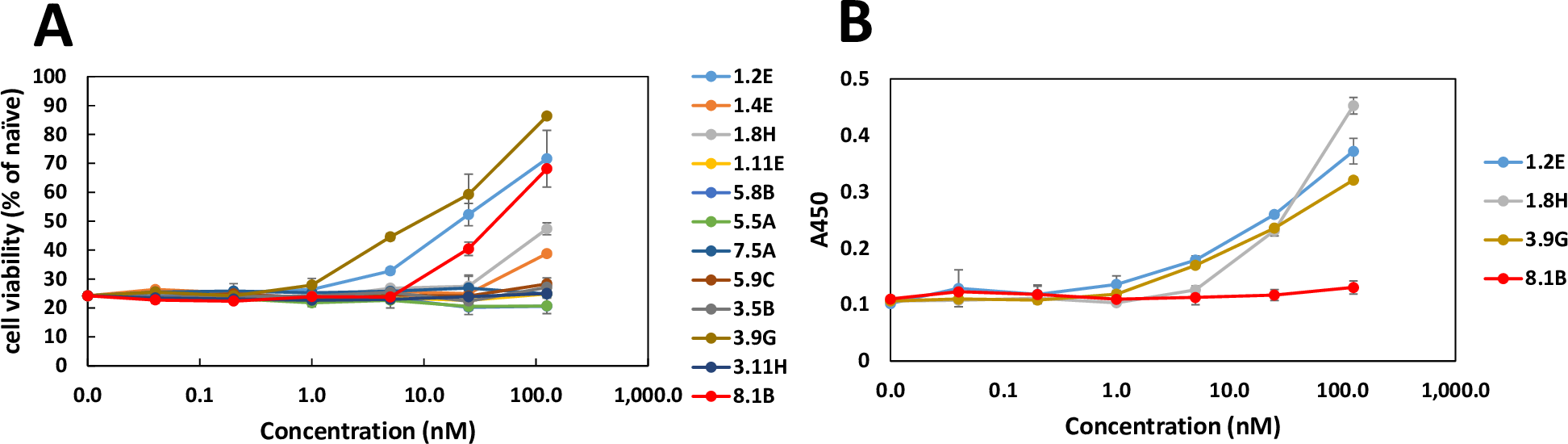

Supplement: S3 Fig — (A) Monomeric DARPins show reduced activity against TcdBUK1 in Vero cells at nanomolar concentrations. IMAC-purified DARPins were added to Vero cells (1.5 × 10^3^ cells/well) together with TcdB toxin (5 pg/mL). Cell viability was quantified 72 hours later by the CellTiterGlo assay and normalized to naïve Vero cells. Error bars represent the standard deviation of 2 independent experiments done in duplicate for samples showing anti-TcdB activity. (B) Relative binding of selected DARPins to UK1 TcdB was determined using ELISA. Serially diluted DARPins were added to microtiter plates coated with 4 μg/mL of TcdB. Results are representative of 2 independent experiments. DARPin, designed ankyrin repeat protein; ELISA, enzyme-linked immunosorbent assay; IMAC, Immobilized metal affinity chromatography; TcdB, C. difficile toxin B. (TIF) [file pbio.3000311.s003.tif]

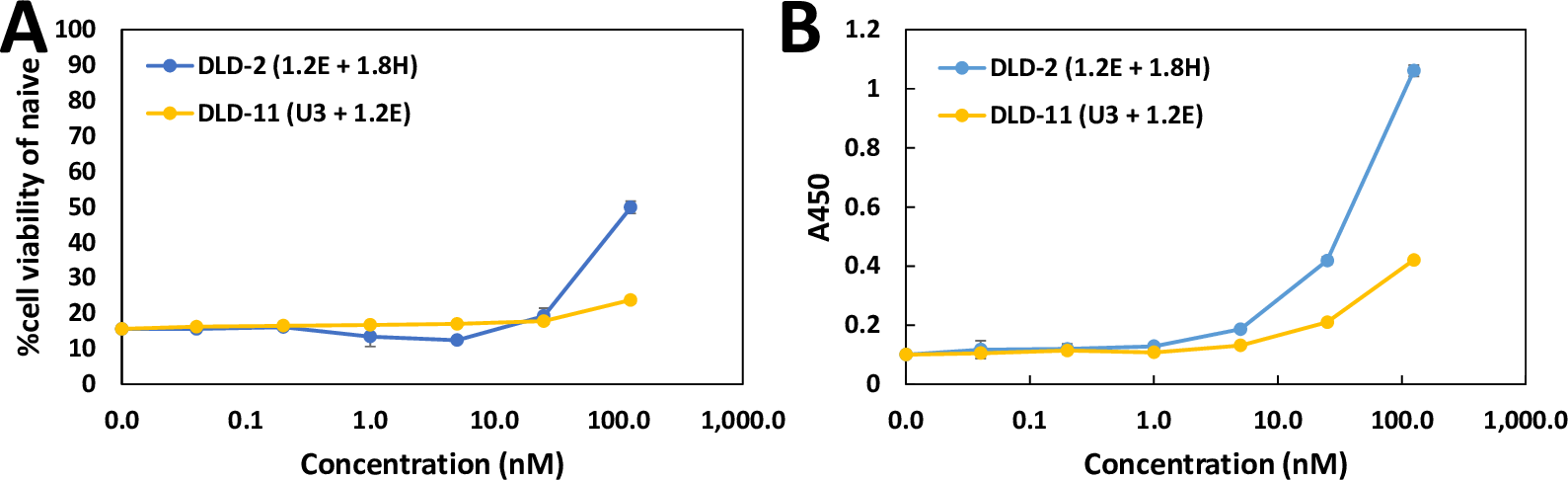

Supplement: S4 Fig — (A) Dimeric DARPins show reduced activity against UK1 TcdB in Vero cells at nanomolar concentrations. IMAC-purified DARPins were added to Vero cells (1.5 × 10^3^ cells/well) together with TcdB (5 pg/mL). Cell viability was quantified 72 hours later by the CellTiterGlo assay and normalized to naïve Vero cells. Error bars represent the standard deviation of triplicate samples. (B) Relative binding of selected dimeric DARPins to UK1 TcdB was determined using ELISA. Serially diluted DARPins were added to microtiter plates coated with 4 μg/mL of TcdB. Results are representative of 2 independent experiments. DARPin, designed ankyrin repeat protein; ELISA, enzyme-linked immunosorbent assay; IMAC, Immobilized metal affinity chromatography; TcdB, C. difficile toxin B. (TIF) [file pbio.3000311.s004.tif]

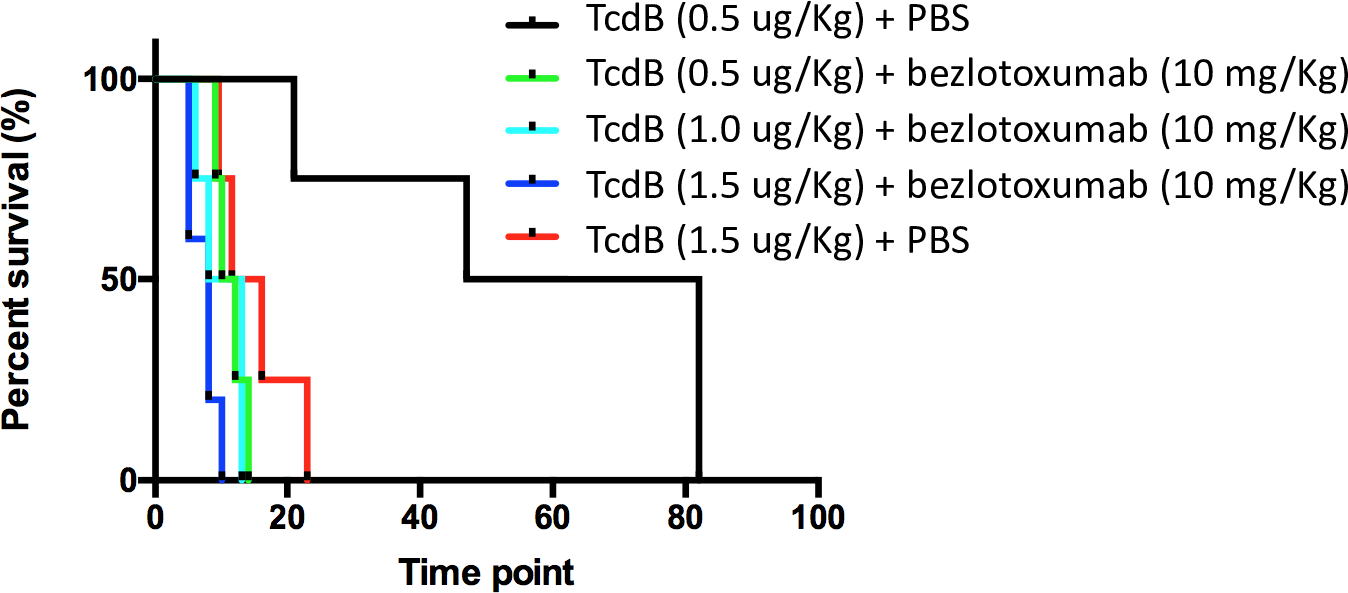

Supplement: S5 Fig — IP, intraperitoneally; TcdB, C. difficile toxin B. (TIF) [file pbio.3000311.s005.tif]

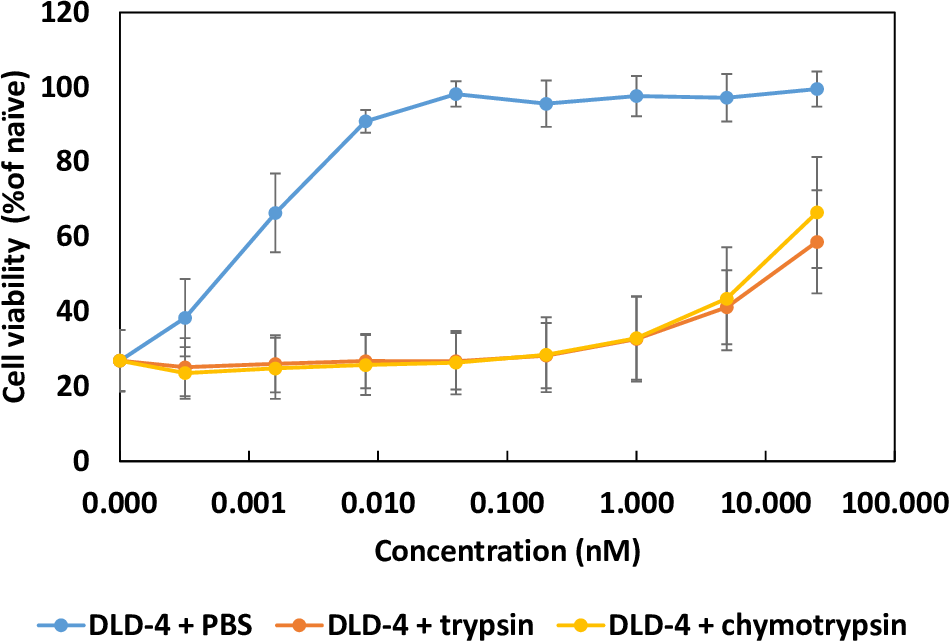

Supplement: S6 Fig — IMAC-purified DARPins were incubated with 1 mg/ml trypsin or chymotrypsin in PBS for 1 hour before being diluted in complete growth medium and added to Vero cells together with TcdB (5 pg/mL). Cell viability was quantified 72 hours later by the CellTiterGlo assay and normalized to naïve Vero cells. Error bars represent the standard deviation of 2 independent experiments done in duplicate. DARPin, designed ankyrin repeat protein;; IMAC, Immobilized metal affinity chromatography; TcdB, C. difficile toxin B. (TIF) [file pbio.3000311.s006.tif]

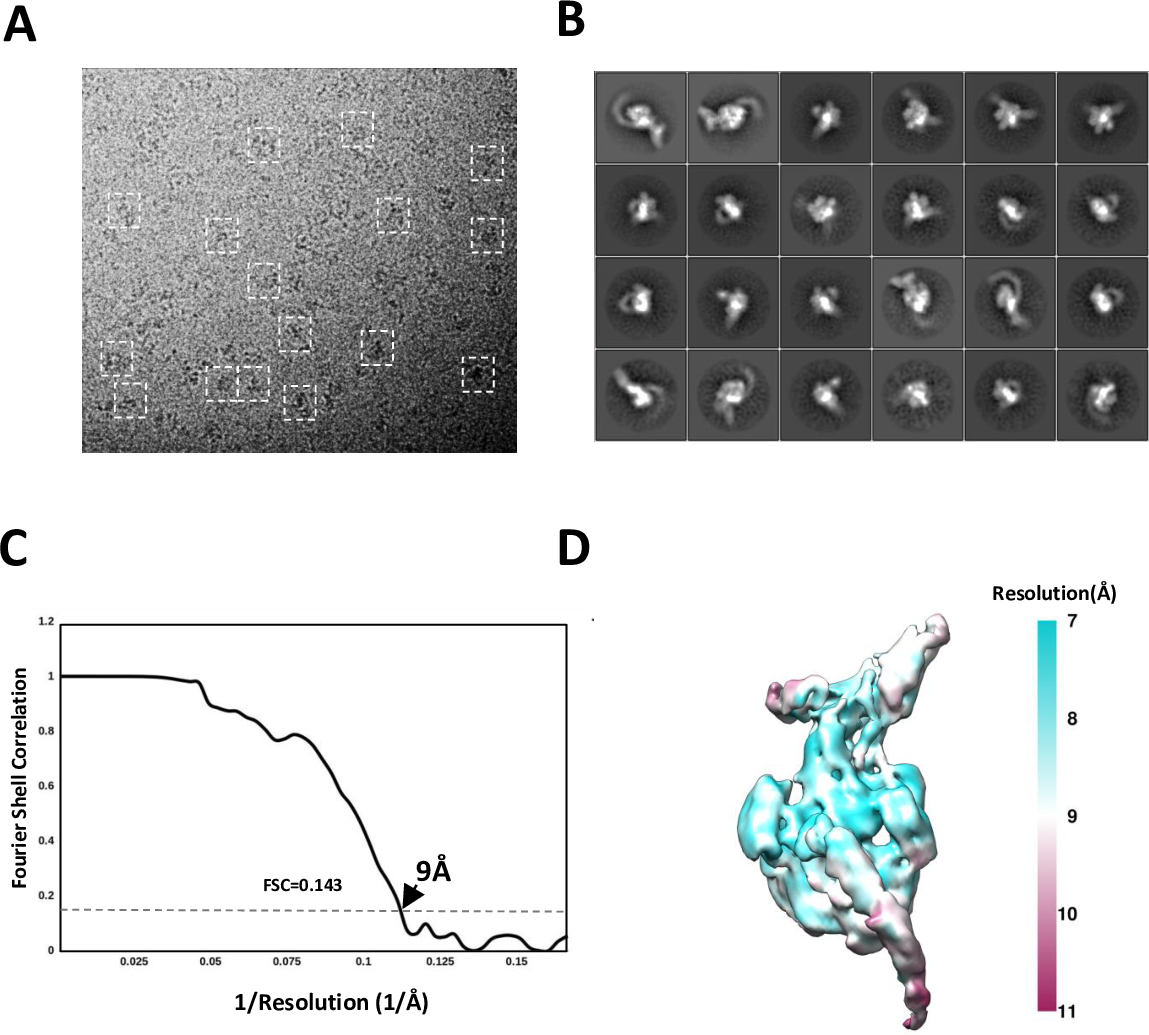

Supplement: S7 Fig — FSC, Fourier Shell Correlation; TcdB, C. difficile toxin B. (TIF) [file pbio.3000311.s007.tif]

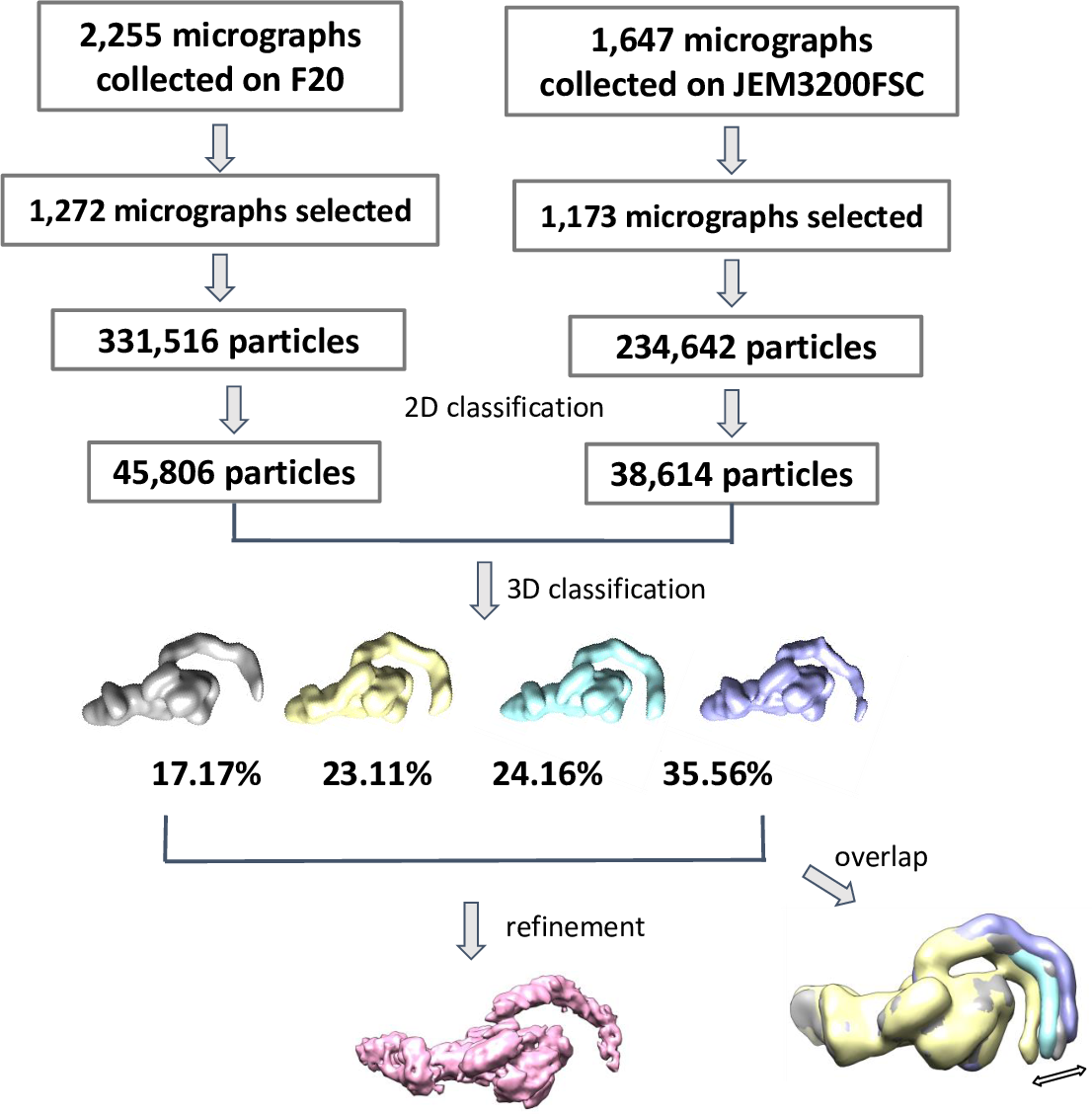

Supplement: S8 Fig — CROPS, combined repetitive oligopeptides; TcdB, C. difficile toxin B. (TIF) [file pbio.3000311.s008.tif]

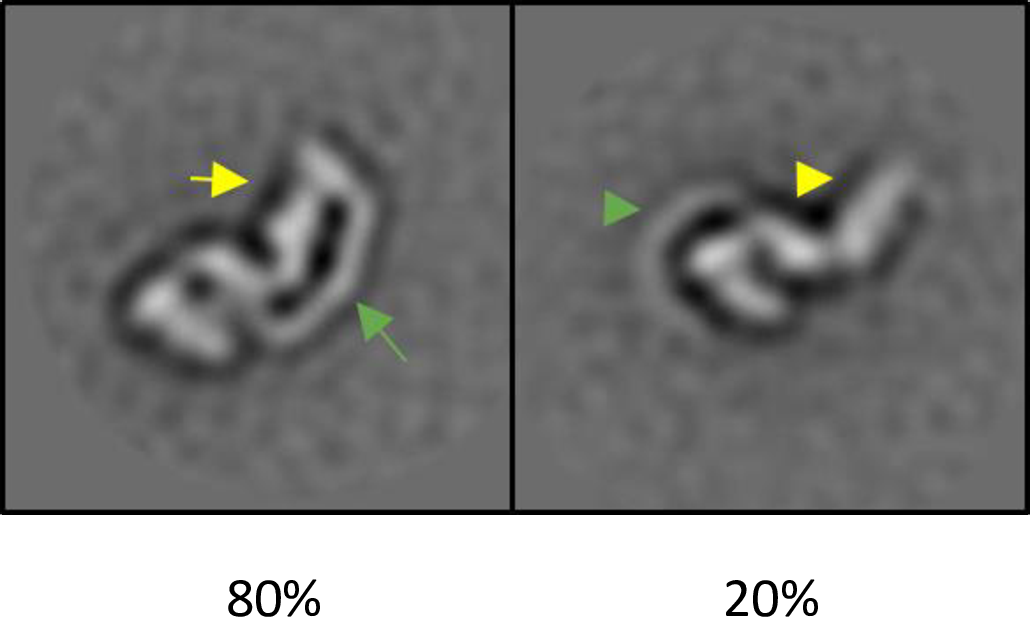

Supplement: S9 Fig — Yellow arrows label the tip of the delivery domain, and green arrows label the CROPS domain. In 80% of the negatively stained particles, the CROPS domain extends toward the delivery domain. This illustrates the problem for the negative-staining EM with this specimen. Only 20% of the data are in a similar conformation as observed in cryo-EM. Purified full-length TcdB (VPI 10463, 0.01 mg/mL) was applied on glow-discharged 400 mesh carbon-coated grids. The sample was stained by immersing in 0.75% uranyl acetate (w/v) for 30 seconds. The prepared grid was loaded and imaged under an FEI Tecnai F20 electron microscope with a field emission gun (FEI Company, the Netherlands) operated at 200 kV, yielding 60 micrographs. Data were collected on a Gatan K2 summit direct detection camera (Gatan, Pleasanton, CA) in the electron-counting mode. A nominal magnification of 19,000 X was used, yielding a pixel size of 1.87 Å. CROPS, combined repetitive oligopeptides; cryo-EM, cryo-electron microscopy; EM, electron microscopy; TcdB, C. difficile toxin B. (TIF) [file pbio.3000311.s009.tif]

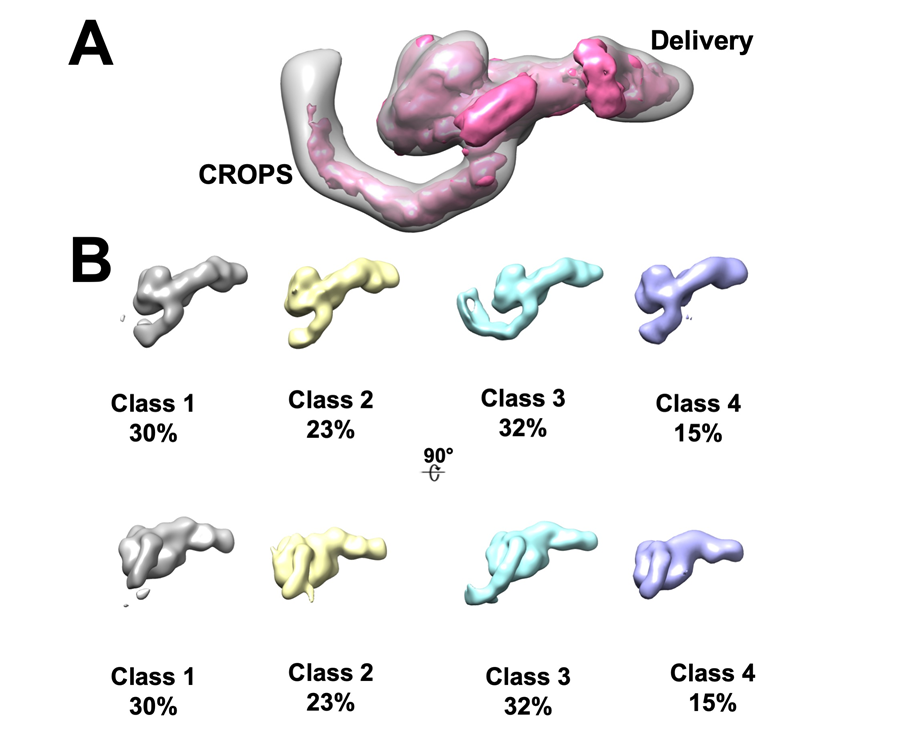

Supplement: S10 Fig — (A) Fitting the cryo-EM map of the DLD-4-bound TcdB (magenta) into cryo-EM map of the apo TcdB (gray transparent) to show the structural similarity between the 2 states. The delivery domain and CROPS domain are indicated. In both states, the CROPS domain protrudes away from the delivery domain. (B) 3D classification of apo TcdB conformations. Side views (top panel) and bottom views (bottom panel) are shown. The orientations of the 4 classes in the side views are the same as in panel A. The number under each class number indicates the percentage of the total number of particles generating that class. CROPS, combined repetitive oligopeptides; cryo-EM, cryo-electron microscopy; TcdB, C. difficile toxin B. (TIF) [file pbio.3000311.s010.tif]

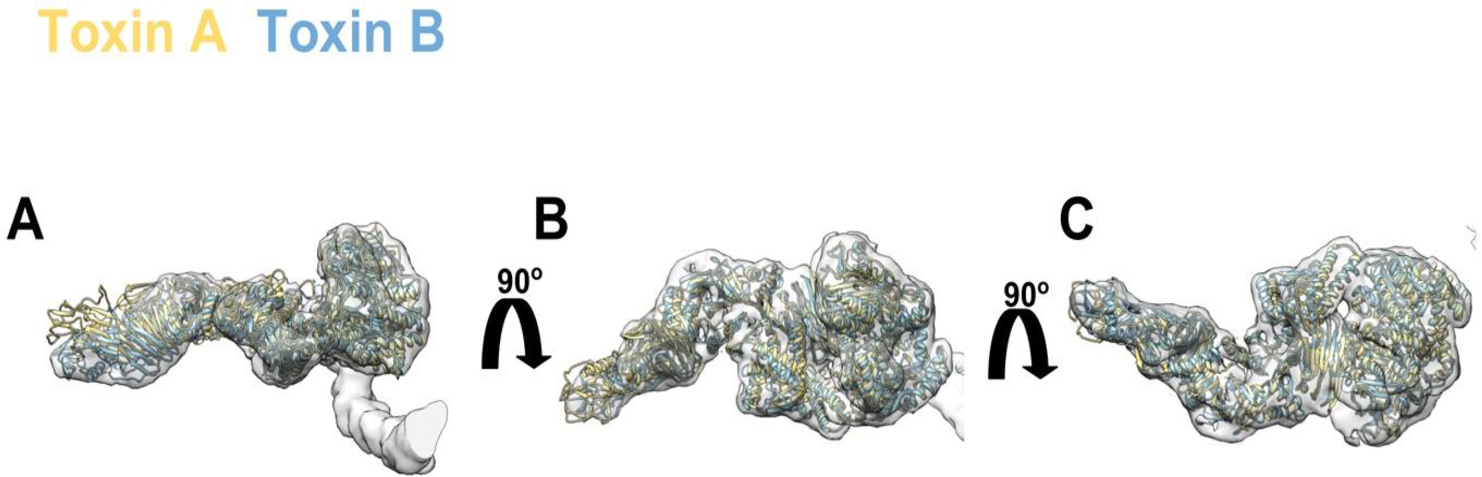

Supplement: S11 Fig — PDB structure of TcdA (4R04) is colored in yellow, and the model of TcdB is colored in blue. Panel B is the view obtained by turning 90 degrees along the horizontal line relative to the view in panel A. Panel C is the view obtained by turning 90 degrees along the horizontal line relative to the view in panel B. PDB, protein data bank; TcdA, C. difficile toxin A; TcdB, C. difficile toxin B. (TIF) [file pbio.3000311.s011.tif]

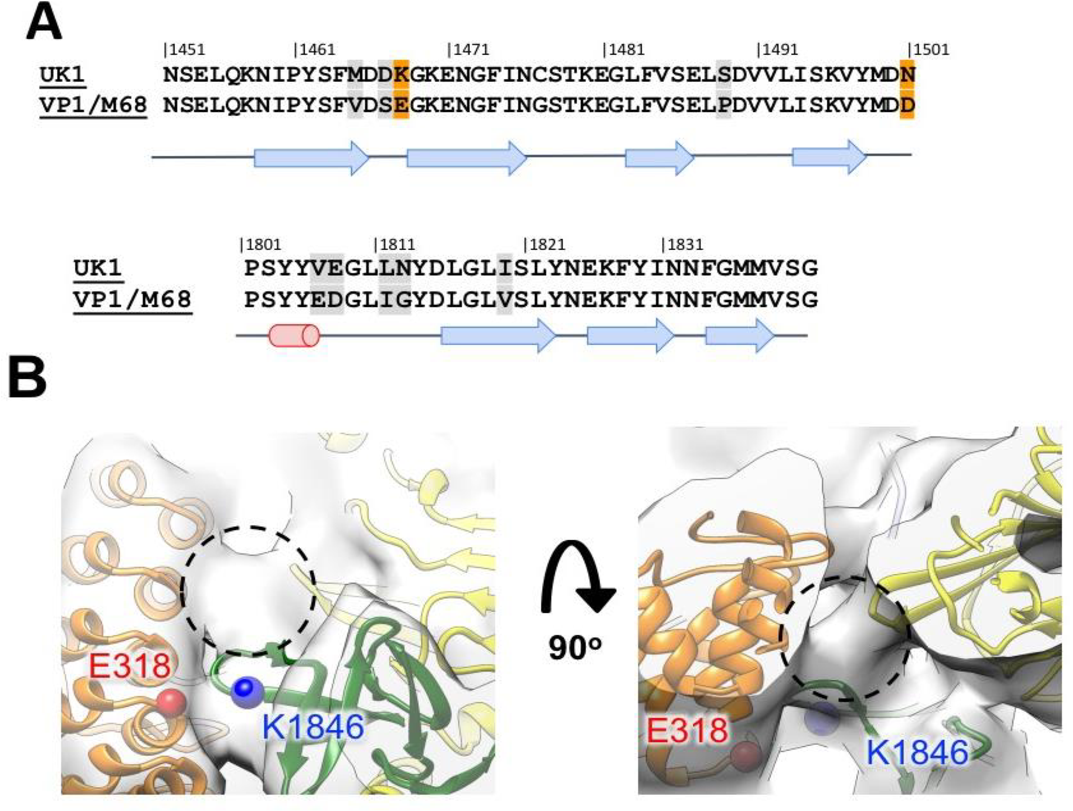

Supplement: S12 Fig — (A) Sequence differences between the TcdB from VPI 10463 and UK1 are labeled gray and orange, with the orange color labeling the residues at the interface of the TcdB and the DARPin. The predicted secondary structure is shown below the sequence. (B) Two views of the β-sheet–like density (within the dashed oval), belonging to residues 1,801 to 1,839, which are currently not modeled. DARPin, designed ankyrin repeat protein; TcdB, C. difficile toxin B. (TIF) [file pbio.3000311.s012.tif]

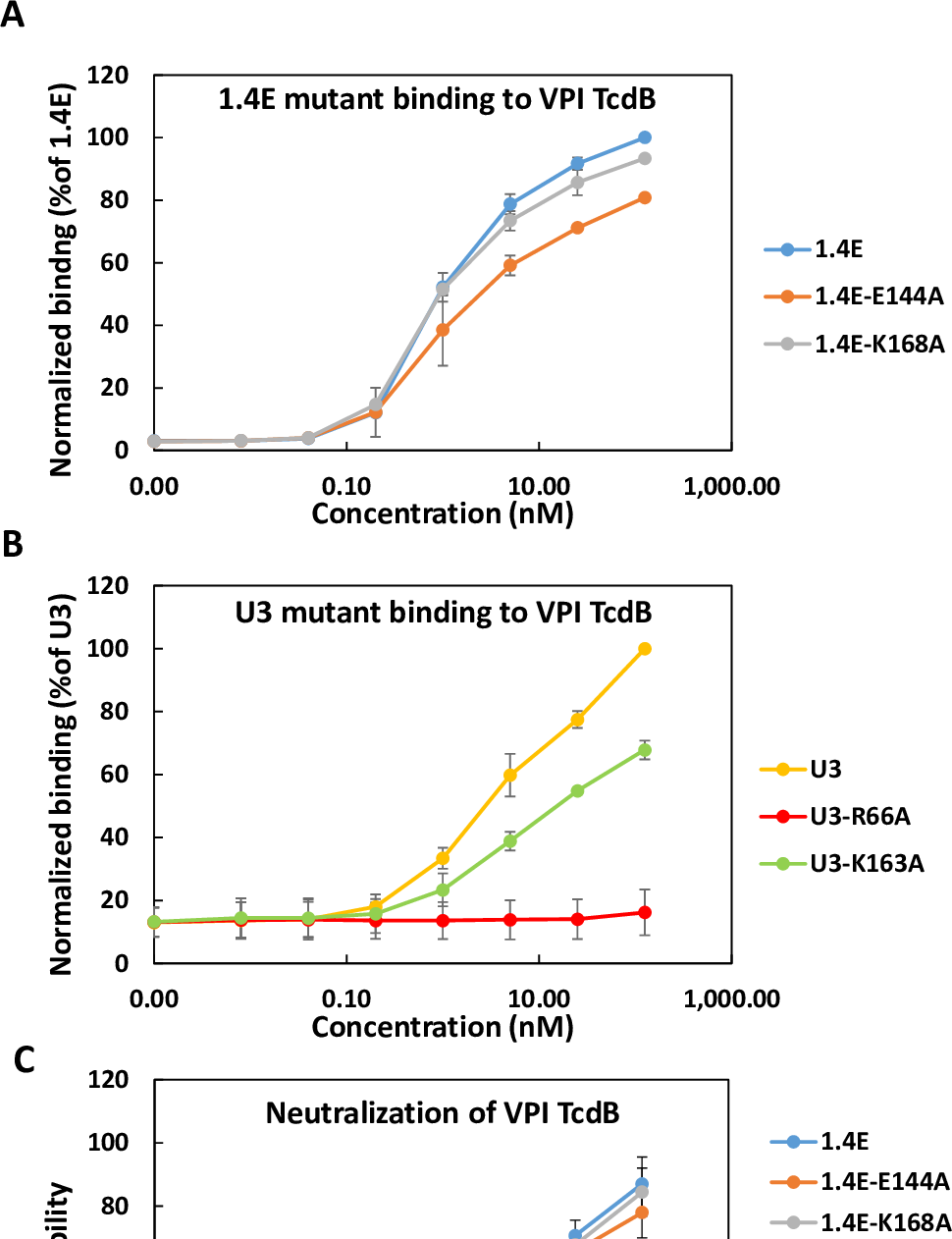

Supplement: S13 Fig — ELISA data of DARPin 1.4E (A) and U3 (B) mutants binding to TcdB from the VPI strain of C. difficile. IMAC-purified DARPins were serially diluted and added to microtiter plates coated with 4 μg/mL of TcdB. ELISA experiment was carried out as described in the Materials and methods section. Data obtained were normalized to values obtained for the unmodified 1.4E and U3. Error bars represent the standard deviation of 2 independent experiments. (C) DARPin 1.4E mutants show similar activity, whereas U3 mutants show reduced activity against VPI TcdB in Vero cells. IMAC-purified DARPins were added to Vero cells (1.5 × 10^3^ cells/well) together with TcdB toxin (5 pg/mL). Cell viability was quantified 72 hours later by the CellTiterGlo assay and normalized to naïve Vero cells. Error bars represent the standard deviation of 2 independent experiments done in duplicate. DARPin, designed ankyrin repeat protein; ELISA, enzyme-linked immunosorbent assay; IMAC, immobilized metal ion affinity chromatography; TcdB, C. difficile toxin B. (TIF) [file pbio.3000311.s013.tif]

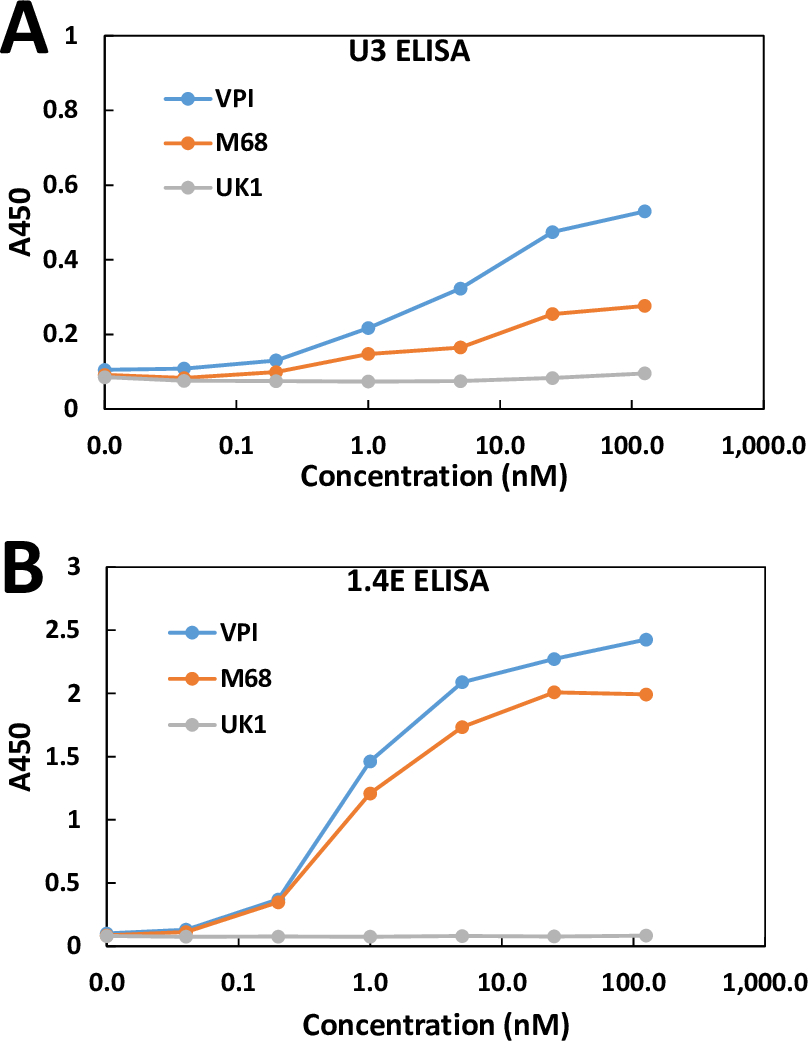

Supplement: S14 Fig — ELISA data of DARPins (A) U3 and (B) 1.4E binding to TcdB from different strains of C. difficile. IMAC-purified DARPins were serially diluted and added to microtiter plates coated with 4 μg/mL of TcdB. ELISA experiment was carried out as described in the Materials and methods section. DARPin, designed ankyrin repeat protein; ELISA, enzyme-linked immunosorbent assay; IMAC, immobilized metal ion affinity chromatography; TcdB, C. difficile toxin B. (TIF) [file pbio.3000311.s014.tif]

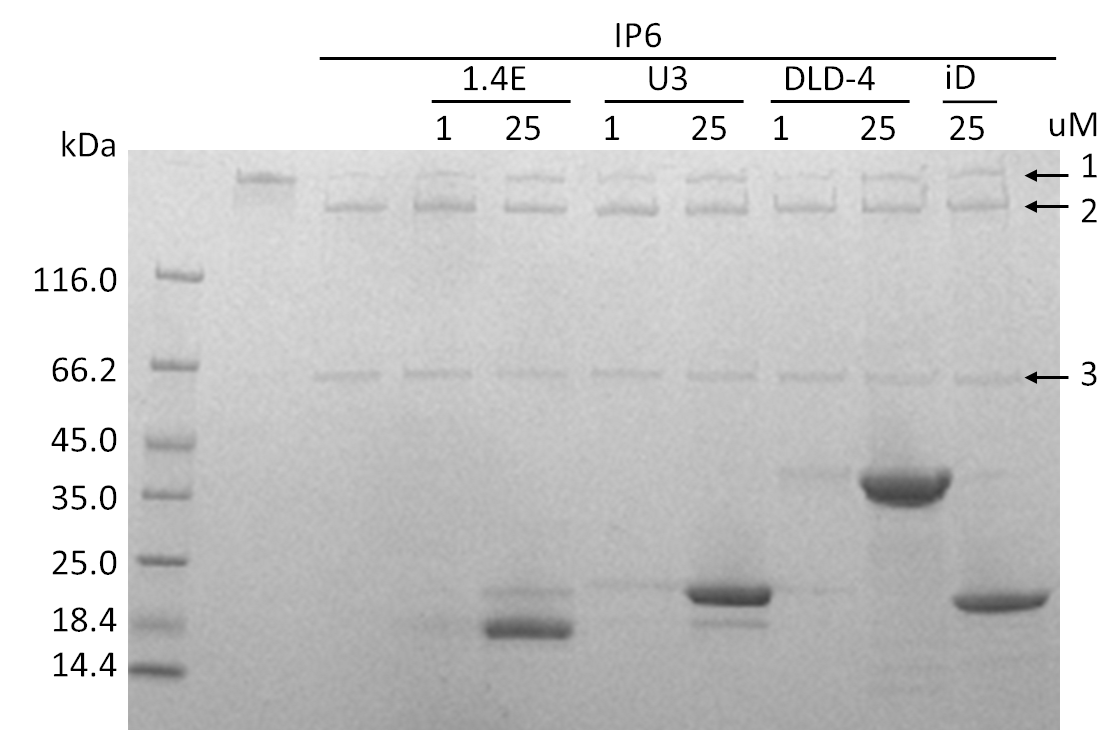

Supplement: S15 Fig — TcdBVPI (20 μM) in tris buffer (20 mM [pH 8]) was incubated with 20 μM IP6 in the absence or presence of the different DARPins at the indicated concentration at 37°C for 1 hour and analyzed on 4% to 20% Mini-PROTEAN TGX gels. (1) Full-length TcdBVPI; (2) C-terminal delivery and CROPS domains; (3) N-terminal GTD domain. iD: an irrelevant DARPin used here as negative control. It appears that a low level of autocleavage inhibition is present at high protein concentration regardless of the identity of the protein. CROPS, combined repetitive oligopeptides; DARPin, designed ankyrin repeat protein; GTD, glucosyltransferase domain; IP6, inositol hexaphosphate; TcdB, C. difficile toxin B. (TIF) [file pbio.3000311.s015.tif]

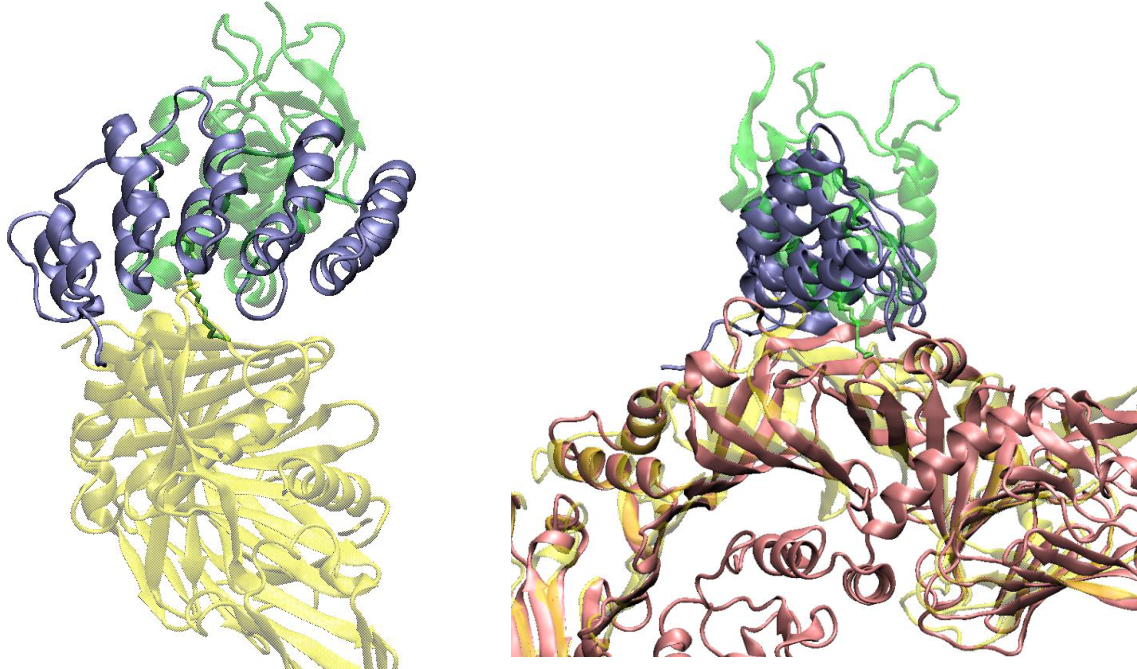

Supplement: S16 Fig — Overlay of the cryo-EM TcdB structure (pink) generated in this work with the TcdB binding domain crystal structure (yellow, PDB 6C0B). Structural alignment indicates complete overlap between FZD2 (green) and U3 (light purple). cryo-EM, cryo-electron microscopy; FZD2, frizzled class receptor 2; PDB, protein data bank; TcdB, C. difficile toxin B. (TIF) [file pbio.3000311.s016.tif]

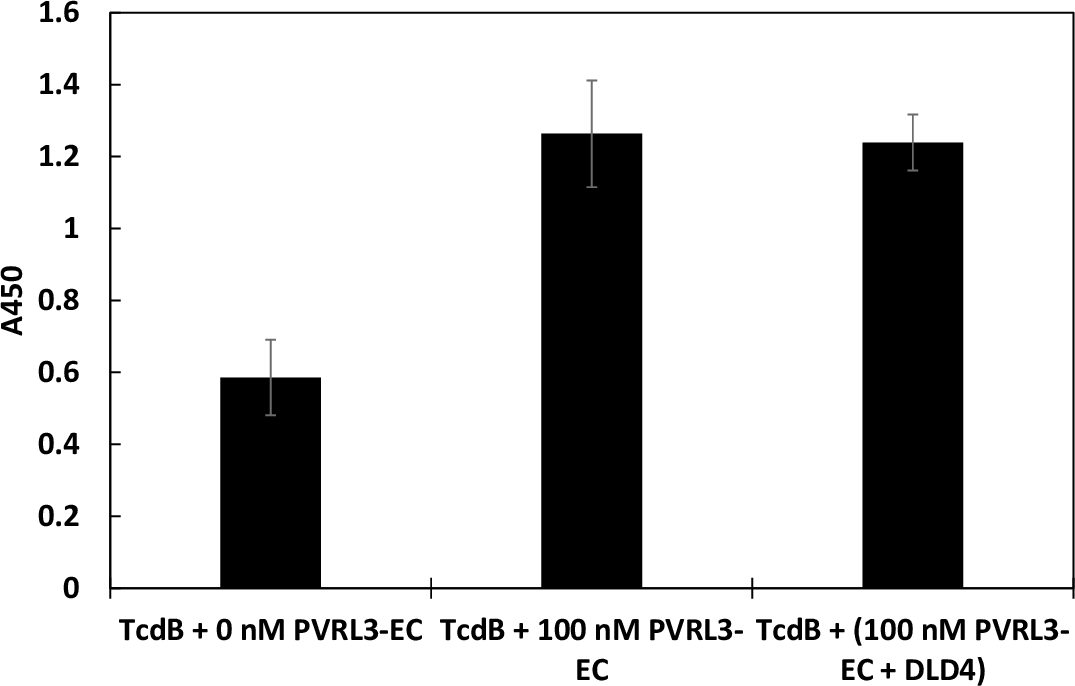

Supplement: S17 Fig — ELISA plates were coated with TcdB followed by treatment with PVRL3-EC alone or mixtures of PVRL3-EC with 250 nM DLD-4. The presence of DLD-4 did not affect the binding of PVRL3-EC to TcdB. Error bars depict the standard deviation of 3 independent experiments. DARPin, designed ankyrin repeat protein; PVRL3-EC, extracellular domain of PVRL3; TcdB, C. difficile toxin B. (TIF) [file pbio.3000311.s017.tif]

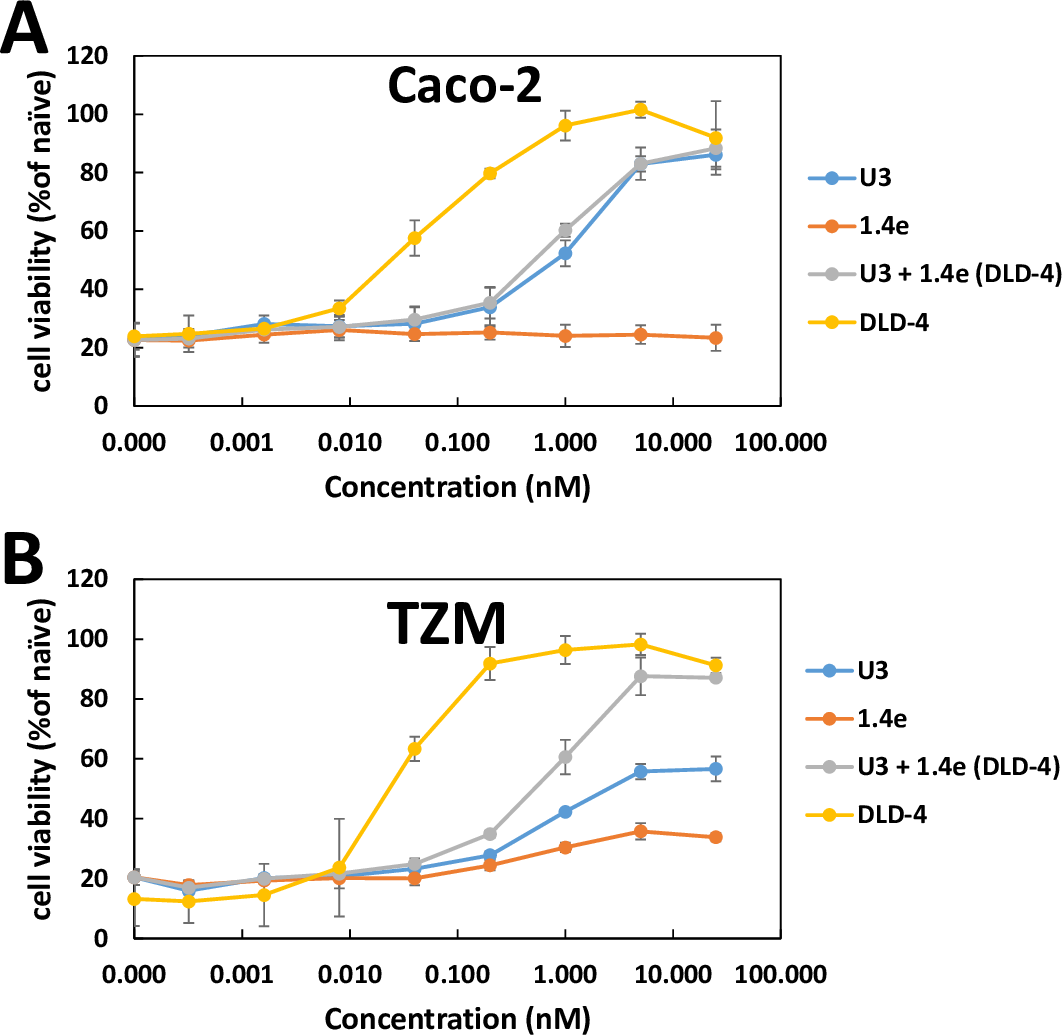

Supplement: S18 Fig — (A) IMAC-purified DARPins were added to Caco-2 cells (1.5 × 10^3^ cells/well) together with 10 pg/mL TcdB. Cell viability was quantified 72 hours later by the CellTiterGlo assay and normalized to naïve Caco-2 cells. Only U3 and DLD-4 inhibited TcdB cytotoxicity in these cells. Error bars represent the standard deviation of 2 independent experiments done in duplicate. (B) IMAC-purified DARPins were added to TZM cells (1.5 × 10^3^ cells/well) together with 5 pg/mL TcdB. Cell viability was quantified 72 hours later by the CellTiterGlo assay and normalized to naïve TZM cells. U3 and 1.4E showed partial inhibition of TcdB in TZM cells. Error bars represent the standard deviation of 2 independent experiments done in duplicate. DARPin, designed ankyrin repeat protein; IMAC, immobilized metal ion affinity chromatography; TcdB, C. difficile toxin B. (TIF) [file pbio.3000311.s018.tif]

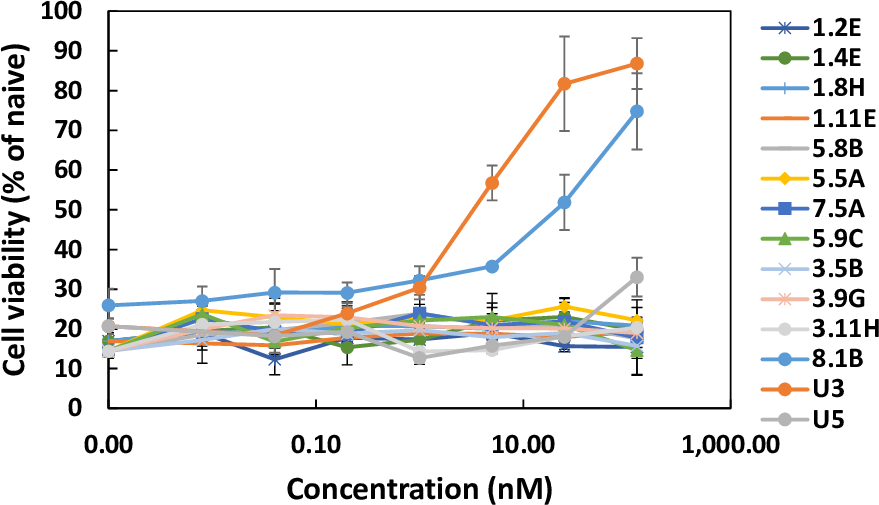

Supplement: S19 Fig — IMAC-purified DARPins were added to Caco-2 cells (1.5 × 10^3^ cells/well) together with TcdB toxin (5 pg/mL). Cell viability was quantified 72 hours later by the CellTiterGlo assay and normalized to naïve Vero cells. Data are representative of 2 independent experiments done in duplicate for DARPins showing anti-TcdB activity. DARPin, designed ankyrin repeat protein; IMAC, immobilized metal ion affinity chromatography; TcdB, C. difficile toxin B. (TIF) [file pbio.3000311.s019.tif]

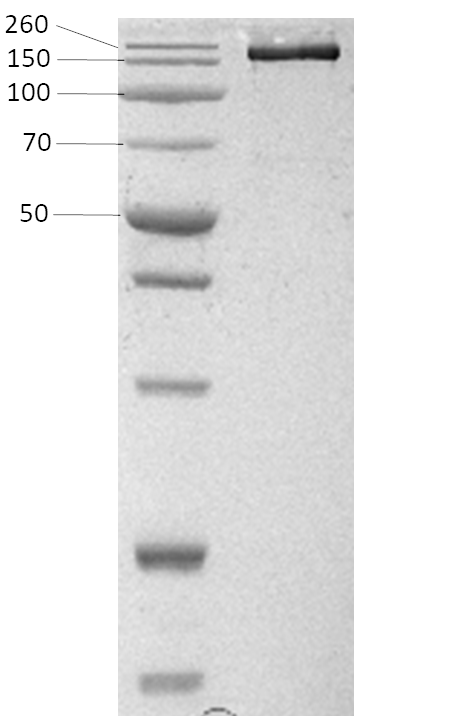

Supplement: S20 Fig — TcdB was expressed and purified as indicated in the Materials and methods section. Purified TcdB (lane 2) was resolved in a 12% poly-acrylamide gel using SDS-PAGE. The gel was stained with Coomassie blue to visualize TcdB. TcdB, C. difficile toxin B. (TIF) [file pbio.3000311.s020.tif]

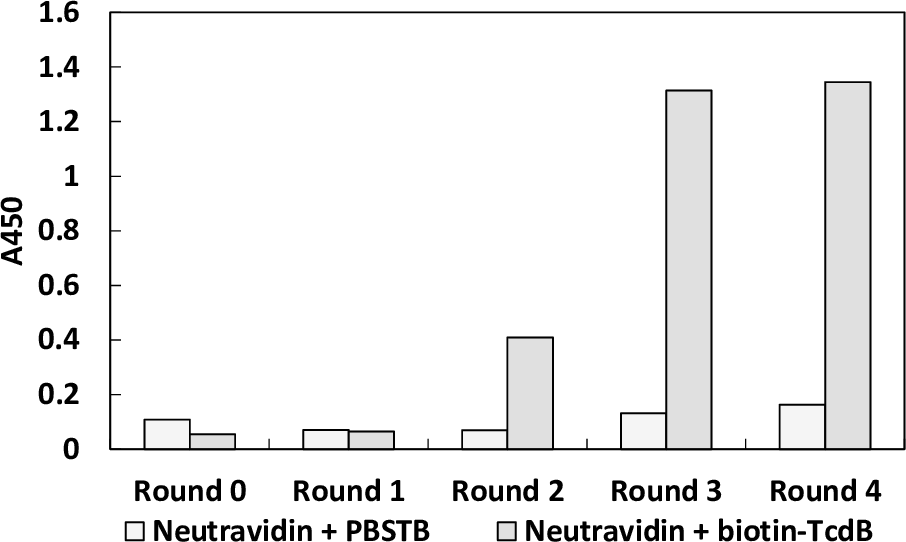

Supplement: S21 Fig — A MaxiSorp 96-well plate was coated with neutravidin (0.2 mg/mL) and then biotin-TcdB. The binding of phage recovered from successive rounds of panning to TcdB was quantified using ELISA. Significant binding was observed after 2 rounds of panning. Binding appeared to plateau after the third round of panning. DARPin, designed ankyrin repeat protein; ELISA, enzyme-linked immunosorbent assay; TcdB, C. difficile toxin B. (TIF) [file pbio.3000311.s021.tif]

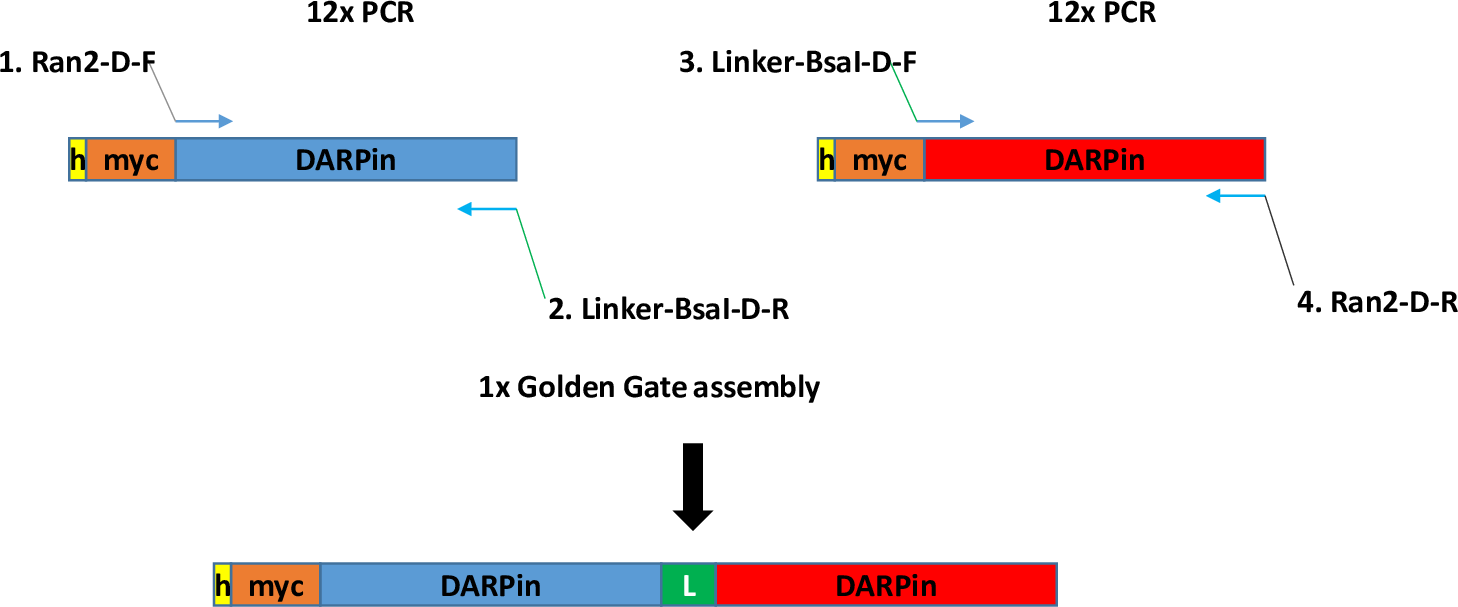

Supplement: S22 Fig — Individual DARPins were amplified using indicated primer sets. Primer Linker-BsaI-D-R added the linker sequence and the type 2 restriction endonuclease BsaI to the 3ʹ end of each DARPin. Primer Linker-BsaI-D-F added the linker sequence and the type 2 restriction endonuclease BsaI to the 5ʹ end of each DARPin. DARPin dimers were then assembled the linker by using a golden gate approach by digestion with BsaI, followed by ligation with T4 DNA ligase. DARPin, designed ankyrin repeat protein. (TIF) [file pbio.3000311.s022.tif]

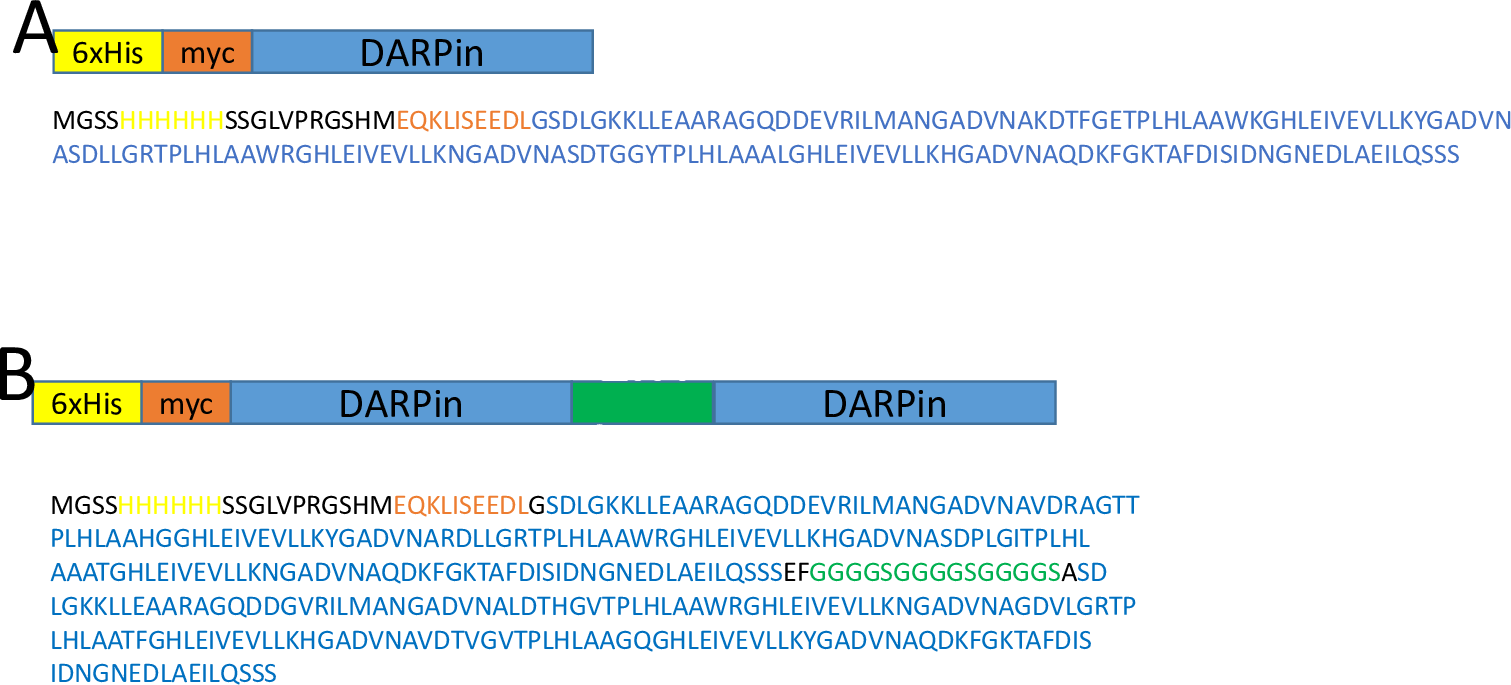

Supplement: S23 Fig — In each DARPin construct there is an N-terminal hexa-His tag followed by an myc tag (A). In the dimeric construct DARPins are separated by a (GGGS)x3 linker sequence (B). DARPin, designed ankyrin repeat protein. (TIF) [file pbio.3000311.s023.tif]

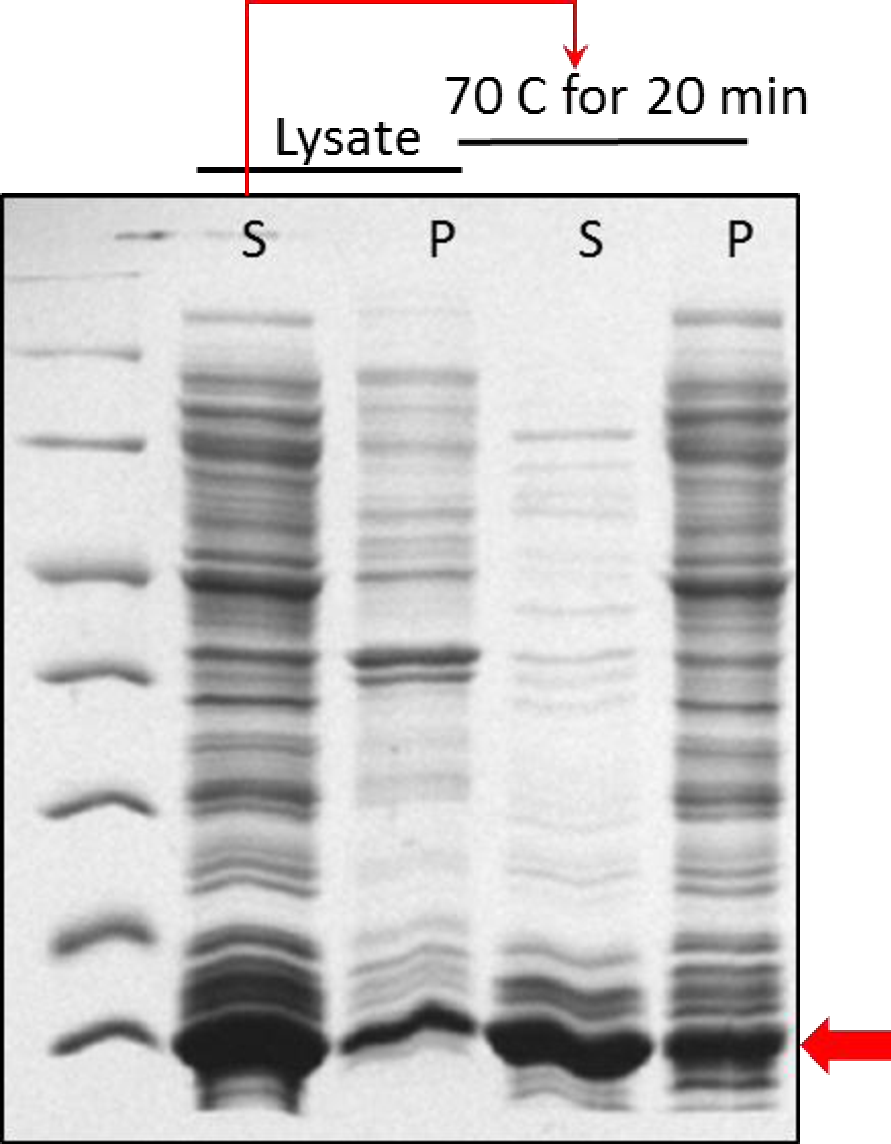

Supplement: S24 Fig — E. coli BL21(DE3) cells transformed with a monomeric DARPin were grown in a well of a 96-well–deep plates (1 mL/well) at 37°C for 8 to 10 hours. Fifty μL of the overnight culture was transferred to fresh plates containing 1 mL/well LB and grown until OD600 approximately 0.6 (approximately 3 hours) prior to the addition of IPTG. The culture was shaken at 400 rpm and at 37°C for 4 hours and was harvested by centrifugation at 1,700g for 20 minutes. The cell pellets were resuspended in 100 μL of PBS supplemented with lysozyme (200 μg/mL), incubated at 37°C for 30 minutes, subjected to 3 cycles of freeze-thaw between −80°C and 37°C and centrifuged at 16,000g for 10 minutes. The soluble fraction was transferred to fresh 96-well deep plates and incubated at 70°C for 20 minutes and centrifuged again, yielding highly enriched DARPin in the supernatant. The red arrow indicates DARPin. DARPin, designed ankyrin repeat protein; P, Pellet; S, Soluble fraction. (TIF) [file pbio.3000311.s024.tif]

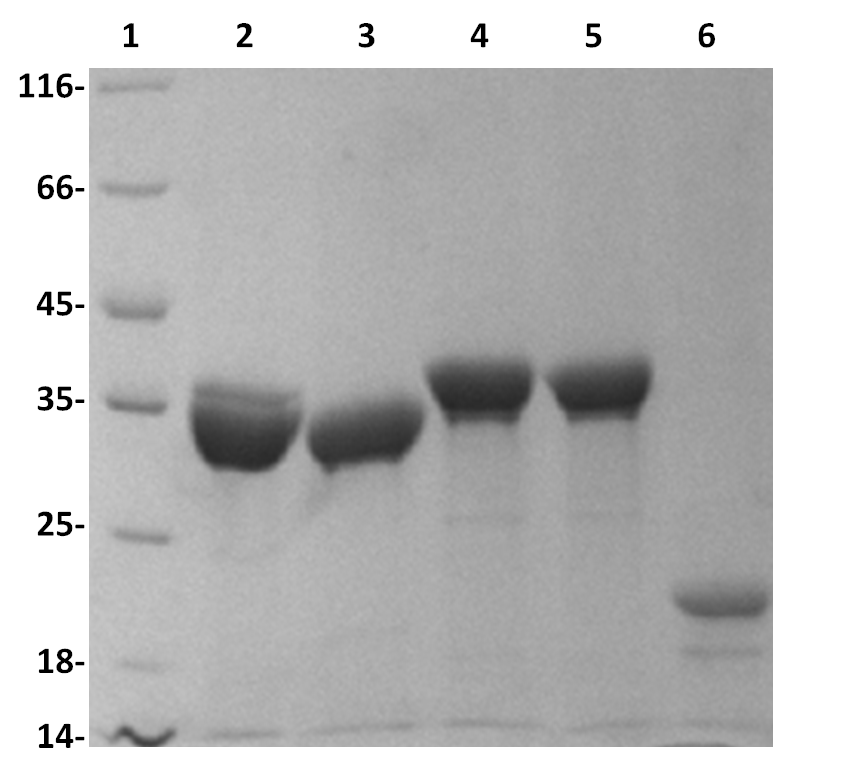

Supplement: S25 Fig — DARPins were expressed and purified as indicated in the Materials and methods section. Selected DARPin dimers (lanes 2–5) and DARPin monomer (lane 6) were resolved in a 12% poly-acrylamide gel using SDS-PAGE. The gel was stained with Coomassie blue to visualize the purified protein. DARPIN, designed ankyrin repeat protein. (TIF) [file pbio.3000311.s025.tif]

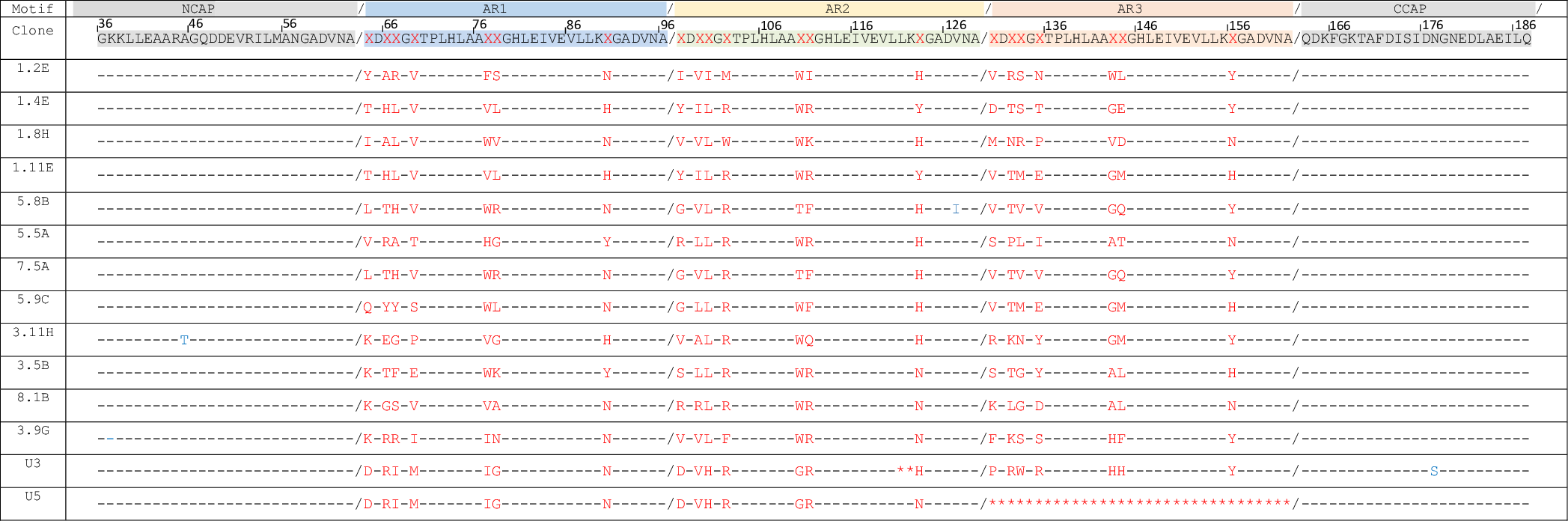

Supplement: S1 Table — DARPIN, designed ankyrin repeat protein. (TIF) [file pbio.3000311.s026.tif]

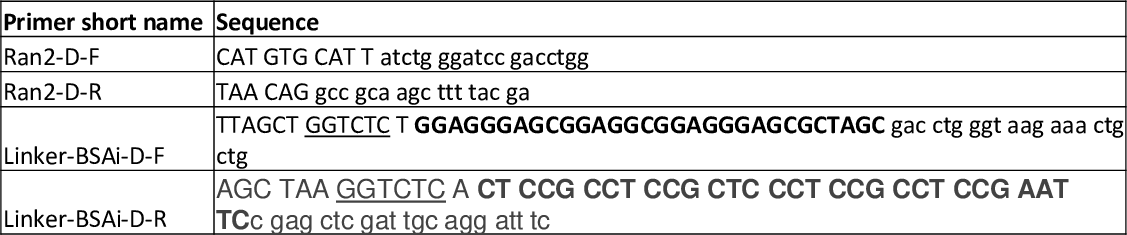

Supplement: S2 Table — Nucleotides binding to individual DARPins are indicated in lowercase. Nucleotides encoding BsaI restriction sites are underlined. Nucleotides encoding the linker sequence are indicated in bolded letters. Primer Ran2-D-F bound to the 5ʹ end of each DARPin. Primer Ran2-D-R binds to the 3ʹ end of each DARPin. Primer Linker-BSAi-D-F binds to the 5ʹ end of each DARPin, adding a linker sequence and the BsaI restriction site to that end. Primer Linker-BSAi-D-R binds to the 3ʹ end of each DARPin, adding a linker sequence and the BsaI restriction site to that end. As such, primer pairs Ran2-D-F and Linker-BsaI-R were used to amplify a single DARPin, adding the linker and the BsaI site to the 3ʹ end. Similarly, primer pairs Ran2-D-R and Linker-BsaI-F were used to amplify a single DARPin, adding the linker and the BsaI site to the 5ʹ end. DARPIN, designed ankyrin repeat protein. (TIF) [file pbio.3000311.s027.tif]
